# Reorganization of metastamiRs in the evolution of metastatic aggressive neuroblastoma cells

**DOI:** 10.1186/s12864-015-1642-x

**Published:** 2015-07-07

**Authors:** Faizan H Khan, Vijayabaskar Pandian, Satishkumar Ramraj, Sheeja Aravindan, Terence S Herman, Natarajan Aravindan

**Affiliations:** Department of Radiation Oncology, University of Oklahoma Health Sciences Center, 940, Stanton L. Young Boulevard, BMSB 737, Oklahoma City, OK 73104 USA; Stephenson Cancer Center, 975 NE 10th Street, BRC 1468, Oklahoma City, OK 73104 USA

**Keywords:** High-risk metastatic disease, Neuroblastoma, MetastamiRS, miRNA, Tumor progression, SH-SY5Y, Aggressive metastatic cells

## Abstract

**Background:**

MetastamiRs have momentous clinical relevance and have been correlated with disease progression in many tumors. In this study, we identified neuroblastoma metastamiRs exploiting unique mouse models of favorable and high-risk metastatic human neuroblastoma. Further, we related their deregulation to the modulation of target proteins and established their association with clinical outcomes.

**Results:**

Whole genome miRNA microarray analysis identified 74 metastamiRs across the manifold of metastatic tumors. RT-qPCR on select miRNAs validated profile expression. Results from bio-informatics across the ingenuity pathway, miRCancer, and literature data-mining endorsed the expression of these miRNAs in multiple tumor systems and showed their role in metastasis, identifying them as metastamiRs. Immunoblotting and TMA-IHC analyses revealed alterations in the expression/phosphorylation of metastamiRs’ targets, including ADAMTS-1, AKT1/2/3, ASK1, AURKβ, Birc1, Birc2, Bric5, β-CATENIN, CASP8, CD54, CDK4, CREB, CTGF, CXCR4, CYCLIN-D1, EGFR, ELK1, ESR1, CFOS, FOSB, FRA, GRB10, GSK3β, IL1α, JUND, kRAS, KRTAP1, MCP1, MEGF10, MMP2, MMP3, MMP9, MMP10, MTA2, MYB, cMYC, NF2, NOS3, P21, pP38, PTPN3, CLEAVED PARP, PKC, SDF-1β, SEMA3D, SELE, STAT3, TLR3, TNFα, TNFR1, and VEGF in aggressive cells *ex vivo* and in a manifold of metastatic tumors *in vivo*. miRNA mimic (hsa-miR-125b, hsa-miR-27b, hsa-miR-93, hsa-miR-20a) and inhibitor (hsa-miR-1224-3p, hsa-miR-1260) approach for select miRNAs revealed the direct influence of the altered metastamiRs in the regulation of identified protein targets. Clinical outcome association analysis with the validated metastamiRs’ targets corresponded strongly with poor overall and relapse-free survival.

**Conclusions:**

For the first time, these results identified a comprehensive list of neuroblastoma metastamiRs, related their deregulation to altered expression of protein targets, and established their association with poor clinical outcomes. The identified set of distinctive neuroblastoma metastamiRs could serve as potential candidates for diagnostic markers for the switch from favorable to high-risk metastatic disease.

**Electronic supplementary material:**

The online version of this article (doi:10.1186/s12864-015-1642-x) contains supplementary material, which is available to authorized users.

## Background

Neuroblastoma (NB), the most common cancer at infancy [1, 2] accounts for about one tenth of pediatric cancer deaths [3–5]. Despite significant advances in understanding the biology of NB and, improved clinical outcomes in the last decade [3, 5], the outcomes for high-risk groups still soar. To that note, cure after relapse of high-risk disease with remarkable heterogeneity, resistance and, poor hematological reserve is extremely rare. Almost half of patients with high-risk NB will relapse with hematogenous metastasis [6] despite intensive multimodal therapy [3, 5, 7–14]. Compared with those with low/intermediate-risk disease (65 %), the five-year overall survival (OS) of patients with high-risk disease is low (<10 %). The rate of long-term survival is even more dismal ten years after diagnosis, with only 2 % OS for patients with stage 4 disease [13, 15]. Cancer cells should successfully complete multiple sequential steps, including spreading from the tumor of origin, intravasation, extravasation and colonization, before they will grow and proliferate at a secondary site and form a new tumor [16, 17]. The capability of cancer cells to metastasize depends on genetic and epigenetic events that are acquired during tumor progression [18]. Despite great advancements in our knowledge of metastasis biology, the molecular mechanisms are still incompletely understood. Remarkably, the regulatory role for miRNAs in metastasis has been established [19–23]. Thus, these miRNAs have been identified as metastamiRs [24], as they have both pro- and anti-metastatic effects. Accordingly, we used a unique, MYCN non-amplified mouse model of human high-risk aggressive metastatic neuroblastoma coupled with whole genome miRNA approach to investigate the functional reorganization of metastamiRs in NB progression.

Genomic amplification of MYCN plays a dominant role in determining the biologic behavior of neuroblastoma and is strongly associated with advanced stage of the disease, rapid tumor progression, therapy resistance and overall poor prognosis [25–29]. However, the realm of MYCN amplification is restricted to about 20 % of all cases of neuroblastoma [26, 30, 31], ~30-40 % of stage 3 and stage 4 and only ~10 % of stage 4 s patients [29, 32]. Critically, the lack of MYCN amplification without either 1p loss or 17q gain further limits the role of MYCN in distinguishing those patients who are likely to survive from those that are destined to fail treatment [33]. Moreover, long-term survival of advanced neuroblastoma patients with MYCN amplification has also been reported [34]. Conversely, prognostic insights and molecular drivers of the MYCN non-amplified high-risk neuroblastoma, that comprises about 60-70 % of stage 3 and stage 4 disease remains unexplored. More importantly, MYCN expression does not correlate with the prognosis of adverse outcome in advanced-stage neuroblastoma with non-amplified MYCN [35]. In this regard, we utilized, MYCN non-amplified cell-line (SH-SY5Y) derived mouse model of human high-risk aggressive metastatic neuroblastoma to define the functional reorganization of metastamiRs in neuroblastoma progression.

miRNAs are endogenous, hairpin-shaped, small non-coding single-stranded RNAs of ^∼^22 nucleotides in length; they serve as post-transcriptional regulators of gene expression [36]. Although miRNAs were initially considered non-functional, recent studies documented the potential of miRNAs to control cell fate [37–39], as well as their conservation across species [40, 41], expression in different tissues and cell types, and their involvement in every biological process. miRNAs function as guide molecules by base pairing with the target mRNA, inducing translation repression or transcripts cleavage [42]. Consequently, miRNA deregulation is a hallmark of several pathological conditions, including cancer.

MetastamiRs are regulatory miRNAs which promote or suppress various steps in the migration and metastasis of cancer cells [24]. It seems that these metastasis-associated miRNAs do not influence primary tumors in either the development or initiation steps of tumorigenesis, but they regulate key steps in the metastatic program and processes, such as EMT, apoptosis, and angiogenesis. Emerging evidence continually recognizes tumor-specific metastamiRs in many tumor models, including breast, lung, prostate, colorectal, gastric, and head and neck cancer. However, in the context of neuroblastoma, no metastamiRs have been grouped or identified. Altered expression of select miRNAs (http://mircancer.ecu.edu) has been shown to exert a causal role in metastasis [20, 22, 23, 43–51]. It is noteworthy that these studies focus on a single miRNA manipulation approach, underscoring the metastatic response through a single gene target. Considering the complexity of sequential steps involved in metastasis, it is necessary to ascertain all causal metastamiRs that play crucial roles in NB metastasis. To our knowledge, this is the first of such an endeavor in the neuroblastoma setting. The results of this study comprehensively identified 74 metastamiRs from whole genome miRNA profiles of a manifold of metastatic tumors from a unique clinically translatable mouse model of aggressive high-risk NB. Further, this study determined the translation of these metastamiR reorganizations into the functional downstream response, that is, target proteins translation that defines metastasis, and the influential role of metastimiRs in clinical outcomes.

## Results

### Spontaneous and reproducible high-risk metastatic disease *in vivo*

**Fig. 1:**
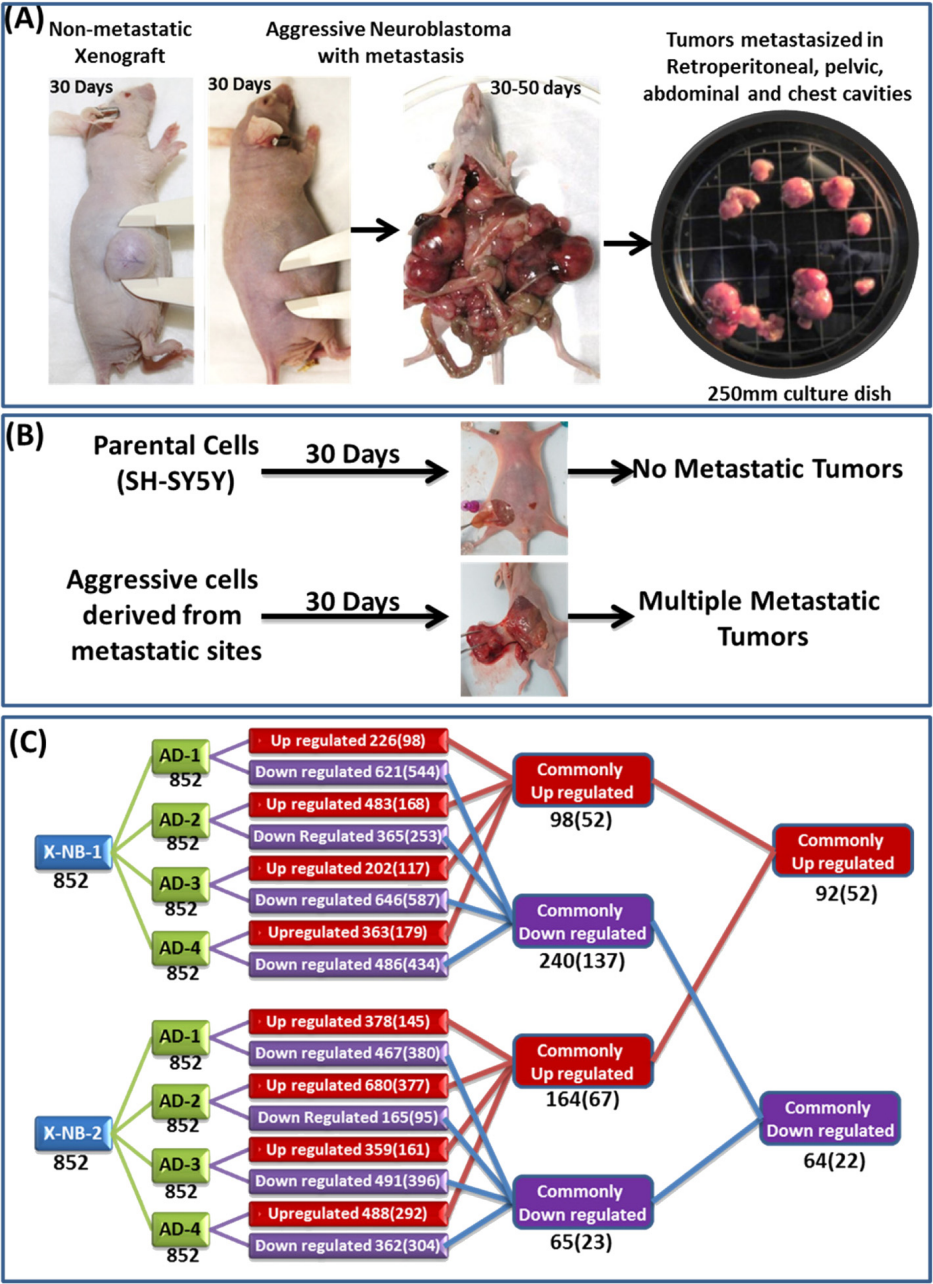
Comparative miRNA profiling in non-metastatic primary xenografts and the manifold of metastatic tumors from animals bearing aggressive neuroblastoma. **a** Representative mice showing non-metastatic xenograft and high-risk aggressive metastatic disease. Plate showing tumors harvested from multiple metastatic sites. **b** Schematic representation showing relative tumorigenic capacity and aggressive disease formation of parental SH-SY5Y and metastatic site derived aggressive cells (MSDACs). Subcutaneously injecting MSDACs produced large xenograft with multiple metastatic tumors in retroperitoneal, pelvic, abdominal, and chest cavities. **c** Traverse analysis of whole genome miRNA expression between non-metastatic (X-NB) and aggressive disease (AD) animals. Total of 852 miRNAs were compared between groups and their alterations are color coded (Red – upregulated; Blue – down regulated). Total number of altered molecules under each comparison is provided in the corresponding box. Numbers in the parenthesis are the molecules that are significantly (> or < 2 fold) modulated

Human neuroblastoma cell-line, SH-SY5Y is the third sub-line of SK-N-SH (SK-N-SH → SH-SY → SH-SY5 → SH-SY5Y) that contains both neuroblast-like (N-type) floating and substrate-adherent (S-type) epithelial-like cells [52]. More importantly, SH-SY5Y cells are unique MYCN non-amplified cells. Xenotransplantation of SH-SY5Y cells resulted in the development of ~200 mm^3^ xenografts in ~70 % of the animals within 30 days (Fig. 1a) [53, 54]. About 30 % of the mice that received identical clones initially (within 10–20 days) showed xenograft development, then subsided to a residual tumor (Fig. 1a). However, over an extended 50–60 day period, these mice suddenly gained weight and presented with multiple clinically-mimicking metastatic tumors in the mediastinum and retroperitoneal, pelvic, abdominal, and chest cavities (Fig. 1a). These mice generally produced 5–12 large, viable, often multi-lobular tumors in multiple sites with well-organized blood supplies (Fig. 1a). This aggressive disease with metastatic dissemination developed over a short period (1–2 weeks) and vigorously, as evidenced by time-lapse non-invasive fluorescence imaging. Parallel xenotransplanted animals with no tumor dissemination to distant sites over the extended period served as the non-metastatic controls.

Metastatic Site Derived Aggressive cell (MSDAC) clones derived from the manifold of metastatic tumors were discretely characterized by karyotyping, whole genome array CGH analysis, and tumorosphere-forming capacity (data reported elsewhere). MSDACs are relatively small and spherical with thin neurites. Administration of MSDACs produced >500 mm^3^ tumors (wet weight 2.40 ± 1.30 g), compared with the <150 mm^3^ xenografts (0.20 ± 0.04 g) resulting from parental cells within 30 days (Fig. 1b). The mice that received MSDACs presented with multiple metastatic tumors in the retroperitoneal, pelvic, abdominal, and chest cavities, demonstrating the reproducibility of the aggressive disease. Conversely, the mice that received parental cells did not exhibit any distant metastasis.

### Reorganization of metastamiRs in high-risk metastatic disease

**Fig. 2:**
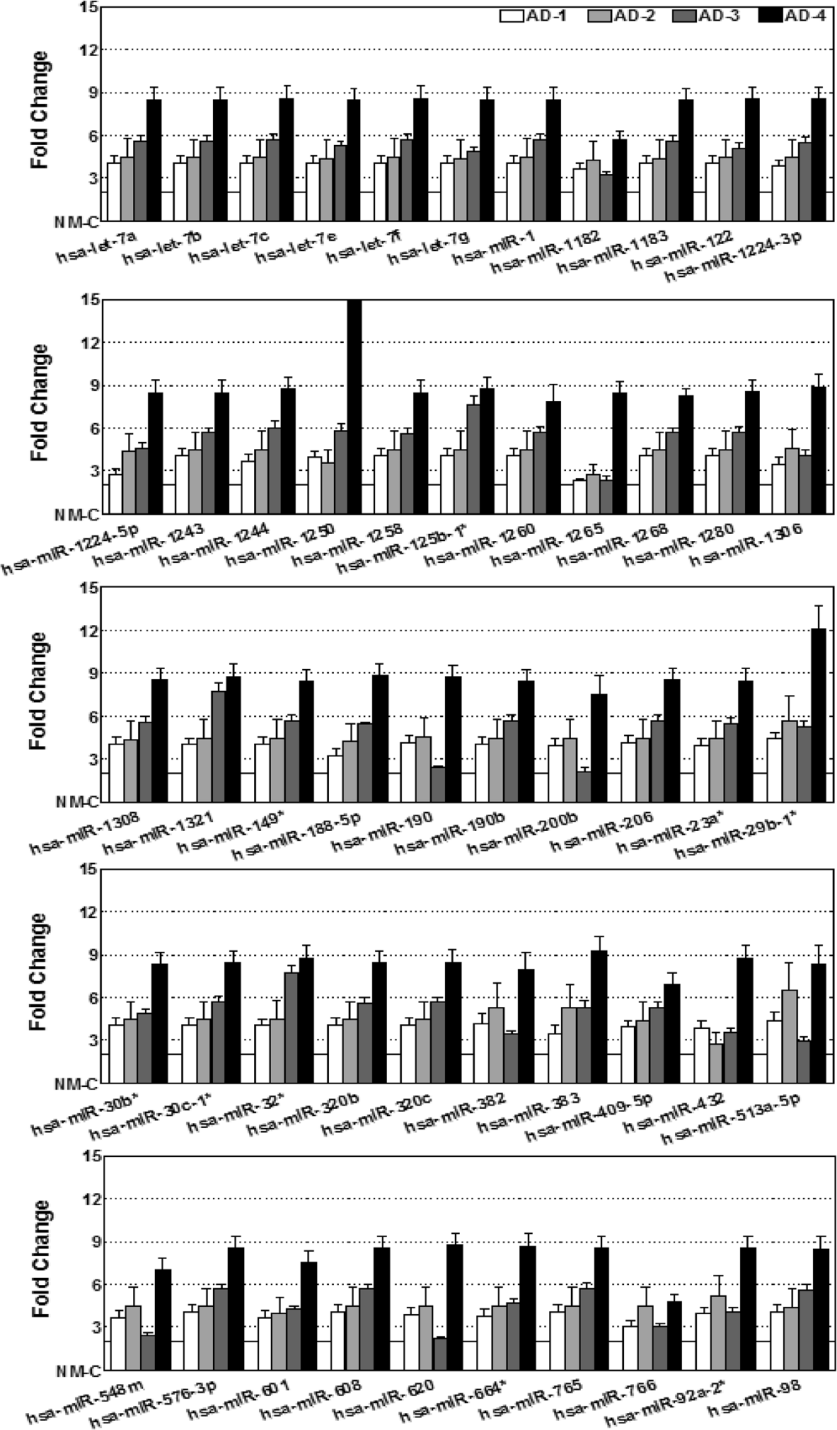
Activated miRNAs in high-risk metastatic neuroblastoma. Histograms showing the expression profile of 52 miRNAs that were significantly (>2 fold) up regulated across the metastatic tumors of the animals with aggressive disease. Data mining was performed using traverse analysis comparing each non-metastatic control to that of the aggressive disease profiles

**Fig. 3:**
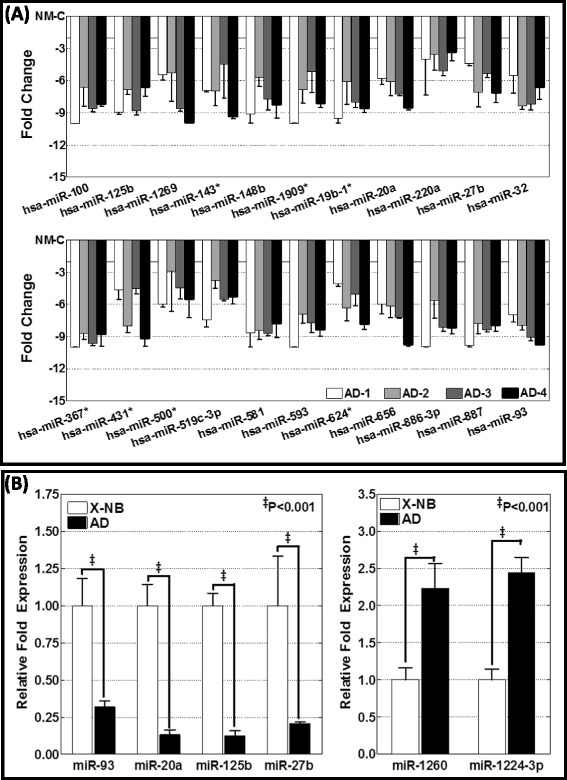
Regulated miRNAs in high-risk metastatic neuroblastoma. **a** Histograms showing the expression profile of 22 miRNAs that were significantly (<2 fold) down regulated across the metastatic tumors of the animals with aggressive disease. Data mining was performed using traverse analysis comparing each non-metastatic control to that of the aggressive disease profiles. **b** miRNA RT-qPCR analysis showing the complete regulation of Hsa-miR-93, Hsa-miR-20a, Hsa-miR-125b and Hsa-miR-27b and, significant increase in the expression of Hsa-miR-1260 and Hsa-miR-1224-3p in metastatic tumors as compared to the non-metastatic xenograft

To define the miRNAs’ modulations in the evolution of high-risk neuroblastoma and to identify crucial players (metastamiRs) that orchestrate metastasis, we adopted a whole genome miRNA profiling approach. A total of 852 functional transcripts were assayed in triplicate for each condition. Traverse analysis between the non-metastatic controls to the manifold of metastatic tumors not only recognized unique animal/tumor-specific expression signatures, but also identified clusters of miRNAs that were commonly up-or downregulated (Fig. 1c). Overall, of the 852 transcripts analyzed, we observed an upregulation of 226, 483, 202, 363 (when compared with X-NB-1), 378, 680, 359, and 488 (compared with X-NB-2) miRNAs in metastatic tumors. Applying stringent criteria (>2 fold), a total of 98, 168, 117, 179 (*vs.* X-NB-1), 145, 377, 161, and 292 (*vs.* X-NB-2) miRNAs were significantly upregulated in the metastatic tumors. Evidently, 52 (*vs.* X-NB-1) and 67 (*vs.* X-NB-2) miRNAs were upregulated across all the metastatic tumors investigated. Upregulation of 52 miRNAs remained consistent across the metastatic tumors, irrespective of the comparisons to the non-metastatic controls (Fig. 2). Conversely, a total of 621, 365, 646, 486 (*vs.* X-NB-1), 467, 165, 491, and 362 (*vs.* X-NB-2) miRNAs were downregulated in metastatic tumors. Significantly (>2 fold), 544, 253, 587, 434 (*vs.* X-NB-1), 380, 95, 396, and 304 (*vs.* X-NB-2) miRNAs were completely suppressed in the metastatic tumors. More importantly, 137 (*vs.* X-NB-1) and 23 (*vs.* X-NB-2) miRNAs were downregulated across all metastatic tumors. Remarkably, downregulation of 22 miRNAs persisted across the metastatic tumors, irrespective of the traverse comparisons to the controls (Fig. 3a).

To validate the altered expression levels observed with whole genome miRNA array, we examined the expression levels of select miRNAs including hsa-miR-1260, hsa-miR-1224-3p (showing significant upregulation; see Fig. 2), hsa-miR-93, hsa-miR-20a, hsa-miR-125b, hsa-miR-27b (showing significant downregulation; see Fig. 3a), using individual miRNA QPCR analysis. Compared to the non-metastatic xenograft, we observed a complete (*P* < 0.001) decrease in the expression of hsa-miR-93, hsa-miR-20a, hsa-miR-125b, and hsa-miR-27b (Fig. 3b). Conversely, we observed a significant (*P* < 0.001) upregulation of hsa-miR-1260 and hsa-miR-1224-3p in metastatic tumor compared with the non-metastatic control (Fig. 3b). These data are consistent with the observed expression levels of these miRNAs using the whole genome approach.

### Altered expression of the target (metastasis-related) proteins validates the translation of the functional response of the neuroblastoma metastamiRs

**Fig. 4:**
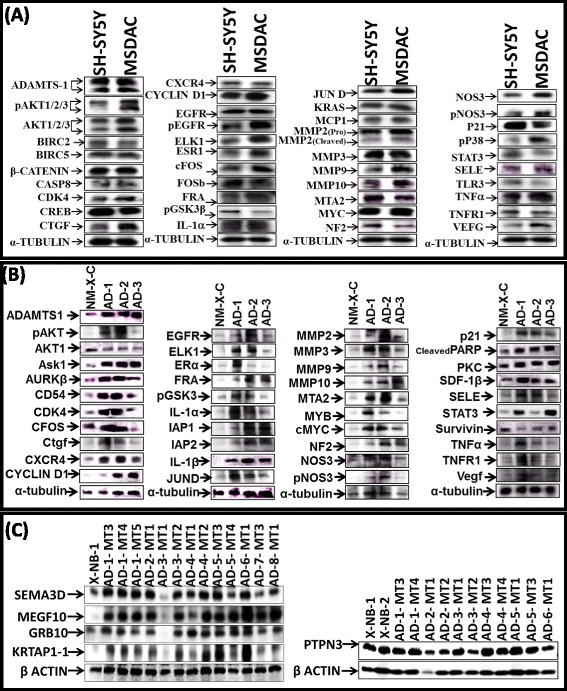
Modulation of metastamiRs target proteins in MSDACs *ex vivo* and in metastatic tumors *in vivo*. Immunoblots showing the expressional modulation of key metastatsis and tumor progression related protein targets of the identified metastamiRs in (**a**) parental SH-SY5Y cells *vs* MSDACs under *ex vivo* conditions and; (**b** and **c**) non-metastatic xenograft *vs* manifold of metastatic tumors from aggressive disease animals *in vivo*

To further substantiate our findings, we investigated the expression levels of target proteins of the identified metastamiRs. Since metastasis is a complex processes, and as we identified a total of 74 metastamiRs in this setting, we examined a total of 49 targets that are involved in tumor progression, i.e., metastasis*,* in MSDACs and a manifold of metastatic tumors. MetastamiRs regulating these targets and their expression status in our study are presented in Table 1 (color-coded). In MSDACs grown *ex vivo,* we observed an induced expression of ADAMTS-1, CASP8, CDK4, CTGF, CYCLIN-D1, ELK1, ESR1, CFOS, FRA, IL-1α, JUND, kRAS, MCP1, MMP2, MMP3, MMP9, MMP10, cMYC, SELE, TNFα, and VEGF, and phosphorylation of AKT1/2/3, NOS3, p38, and EGFR compared with the parental SH-SY5Y cells (Fig. 4a). Compared with the non-metastatic xenograft controls, we observed a robust increase in ADAMTS-1, ASK1, AURKβ, Birc1, Birc2, Birc5, CD54, CDK4, CTGF, CXCR4, CYCLIN-D1, EGFR, ELK1, ESR1, CFOS, FRA, GRB10, pGSK3β, IL1α, JUND, KRTAP1-1, MEGF10, MMP2, MMP3, MMP9, MMP10, MTA2, MYB, cMYC, NF2, P21, PTPN3, CLEAVED PARP, PKC, SDF-1β, SEMA3D, SELE, STAT3, TNFα, TNFR1, and VEGF expression, as well as AKT1/2/3 and NOS3 phosphorylation in the manifold of metastatic tumors (Fig. 4b and c).

**Table 1:** Targets analyzed by the western-blot and the expression status (color coded) of the corresponding metastamiRs in high-risk metastatic neuroblastoma

ADAMTS-1	**miR-1224-3p, miR-1260**, *miR-125b*
ASK1	**miR-1258**, **miR-1243**, *miR-519c-3p*, **miR-1224-5p**, *miR-20a*, *miR-93*, **miR-1260**, miR-23a*, miR-30b*
AURK ß	**miR-765**
BIRC2	miR-500*
cMYC	**let-7a**, **let-7b**, **let-7c**, **let-7e**, **let-7f**, **let-7g**, **miR-98**
CASP8	*miR-519c-3p*, miR-143*, *miR-20a*, *miR-93*, **miR-1224-3p**
CD1C	**miR-1306**, **miR-190**, **miR-190b**, **miR-620**
CD54	miR-431*
CDK4	**miR-548m**, **miR-765**
CFOS	**miR-383**
PARP	*miR-519c-3p*, **miR-766**
CREB	**miR-190**, **miR-190b**, **miR-1224-5p**, *miR-20a*, *miR-93*, *miR-125b*, *miR-27b*
CTGF	miR-30c-1*, **miR-576-3p**, **miR-383**, miR-143*, miR-30b*
CXCR4	**miR-1**, **miR-206**
CYCLIN D1	**let-7a**, **let-7b**, **let-7c**, **let-7e**, **let-7f**, **let-7g**, **miR-98**, **miR-383**, *miR-1269*, **miR-1**, **miR-206**, *miR-20a*, *miR-93*, miR-23a*, **miR-608**
EGFR	miR-664*, **miR-1250**, *miR-1269*, **miR-1**, **miR-206**, miR-149*, miR-27b, miR-1909*, **miR-608**
ELK1	**miR-1260**, *miR-125b*, **miR-765**, **miR-1321**, **miR-608**
NOS3	*miR-887*, **miR-1244**, **miR-765**
ESR1	**miR-188-5p**, **miR-190**, **miR-190b**, **miR-1182**, *miR-519c-3p*, **miR-1**, **miR-206,** miR-148b, miR-20a, miR-93, miR-1909*
E-SELECTIN	*miR-593*
FOS B	**miR-766, miR-1, miR-206**, *miR-148b*, miR-1224-3p, miR-1260, miR-23a*, miR-765, miR-27b, miR-1909*, **miR-608**
FRA	*miR-593*, miR-149*
GRB10	*miR-519c-3p*, miR-513a-5p, miR-30b*, *miR-125b*
IL-1α	miR-149*, miR-30b*
JUN D	**miR-1**, **miR-206**, **miR-1321**
**kRAS**	**let-7a, let-7b, let-7c, let-7e, let-7f, let-7g, miR-98, miR-1243,** miR-143*, miR-513a-5p, miR-1, miR-206, miR-200b, miR-30b*, miR-27b
KRTAP1-1	**miR-601, miR-1258,** miR-143*, miR-23a*
MCP1	miR-664*, *miR-593* **, miR-1, miR-206**
MEGF10	**miR-190, miR-190b, miR-1258, miR-383**
MMP10	miR-32*, *miR-32*, miR-367*, *miR-148b*
MMP2	*miR-20a*, *miR-93*, **miR-765**, *miR-125b*, **miR-1321**, **miR-608**
MMP3	*miR-20a, miR-93*
MTA2	*miR-148b*, **miR-1321**
MYB	**miR-1224-5p**
NF2	miR-1909*, miR-32*, *miR-32*, miR-367*, **miR-608**
GSK3B	miR-624*, **miR-1**, **miR-206**, miR-23a*, *miR-27b*
P38	**miR-1258, miR-1243, miR-513a-5p**, *miR-125b, miR-27b*
P21	miR-92a-2*, miR-32*, miR-32, miR-367*, miR-20a, miR-93, **miR-765, miR-608**
AKT2	**miR-200b**, *miR-148b*, **miR-1224-3p**, **miR-1260**, miR-1909*, **miR-608**
AKT3	**miR-320b**, **miR-320c**, miR-149*, *miR-20a*, *miR-93*
PKC	miR-30b*, **miR-1321**, miR-1909*
PTPN3	*miR-593*, *miR-20a*, *miR-93*, **miR-765**, miR-27b
SDF-1ß	miR-624*, **miR-1182**, *miR-519c-3p*, **miR-1260**, miR-23a*, **miR-765**, miR-1909*
SEMA3D	**miR-432**, **miR-1268**, **miR-1321**
STAT3	**let-7a**, **let-7b**, **let-7c**, **let-7e**, **let-7f**, **let-7g**, **miR-98**, **miR-1244**, *miR-20a*, *miR-93*, *miR-125b*, miR-1909*, **miR-608**
SURVIVIN	**miR-1182**, **miR-1321**
TNFR1	**miR-766**
TNF-α	miR-125b-1*, *miR-519c-3p*, miR-1909*
VEGF	**miR-383**, **miR-1**, **miR-206**, **miR-200b**, *miR-20a, miR-125b, miR-27b*

**Up regulated**; *Down Regulated*

**Fig. 5:**
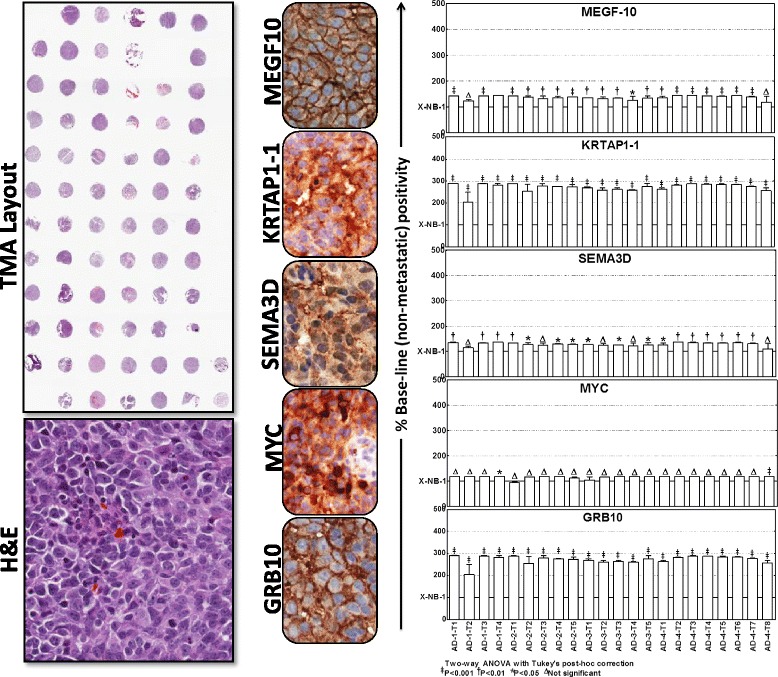
Localization and expressional modulation of metastamiRs target proteins in metastatic neuroblastoma. Representative image of the tissue micro array constructed with the replicates of non-metastatic xenografts controls and the manifold of metastatic tumors from spontaneous aggressive disease as well as the reproduced aggressive disease animals. Automated IHC stained panels showing the staining pattern and cellular localization of the metastamiRs’ protein targets (GRB10, MYC, SEMA3D, KRTAP1-1, MEGF10) in tumor samples. Histograms of Aperio-Spectrum image analysis and quantification of positivity for each target protein analyzed across the metastatic tumors of various animals presented with aggressive disease. The positivity values are compared to the non-metastatic xenograft controls using ANOVA with Tukey’s post-hoc correction using GraphPad PRISM

We then sought to define and typify the target alterations in aggressive neuroblastoma. We used a custom-made TMA constructed with a manifold of tumors from the metastatic sites of several animals coupled with non-metastatic xenograft controls. These were subjected to automated IHC and image analysis for select targets, including MEGF10, KRTAP1-1, SEMA3D, MYC, and GRB10. Consistent with our immunoblotting observations under *ex vivo* and *in vivo* conditions, MEGF10, KRTAP1-1, SEMA3D, MYC, and GRB10 IHC staining revealed relatively strong positivity in metastatic tumors (Fig. 5). Multiple EGF-like-domains-10 (MEGF10) positive staining appeared in brown, and was predominantly localized in plasma membranes in a punctuated pattern (Fig. 5). MEGF10 immunoreactivity was barely detectable in non-metastatic NB xenografts. However, the manifold of metastatic tumors exhibited significantly high immunoreactivity for MEGF10 localization (Fig. 5). Keratin-associated protein family 1–1 (KRTAP1-1) positivity appeared in brown, and was predominantly localized in the cytoplasm (Fig. 5). Consistent with our immunoblotting data, we observed a profound and significant (*P* < 0.001) increase in the expression of KRTAP1-1 in the metastatic tumors as opposed to the non-metastatic controls (Fig. 5). Likewise, Semaphorin-3D (SEMA-3D) IHC exhibited moderate cytoplasmic positivity with a dark brown color (Fig. 5). Compared with the non-metastatic controls, increased expression of SEMA-3D was evident in almost all metastatic tumors analyzed. MYC-IHC revealed strong nuclear positivity in all tumors analyzed. We observed a marginal increase in MYC expression in the metastatic tumors when compared with non-metastatic controls (Fig. 5). Growth factor receptor-bound protein 10 (GRB10) IHC revealed high levels of localization in non-metastatic neuroblastoma tissues. Notably, immunoreactivity was highly intense and significant (*P* < 0.001) in metastatic tumors (Fig. 5). The positive staining of GRB10 appeared in dark brown, and was predominantly localized in the cytoplasm.

These observations were consistent across tumors from the same animal as well as tumors from different animals. We observed similar alterations in the expression of MEGF10, KRTAP1-1, SEMA3D, MYC, and GRB10 in the manifold of distant tumors reproduced from aggressive disease-bearing animals. Aperio image analysis coupled with PRISM stats were used to demonstrate the increased expression of these target proteins in the tumors of animals with aggressive disease, but not in the non-metastatic xenografts (Fig. 5). *Ex vivo* and *in vivo* upregulation of these targets, which are well—documented candidates for metastasis and tumor progression of the metastamiRs that were identified in this study, underscores the translation of the functional response (metastasis) of metastamiRs in this setting.

### Altered miRNAs dependent regulation of functional targets

**Fig. 6:**
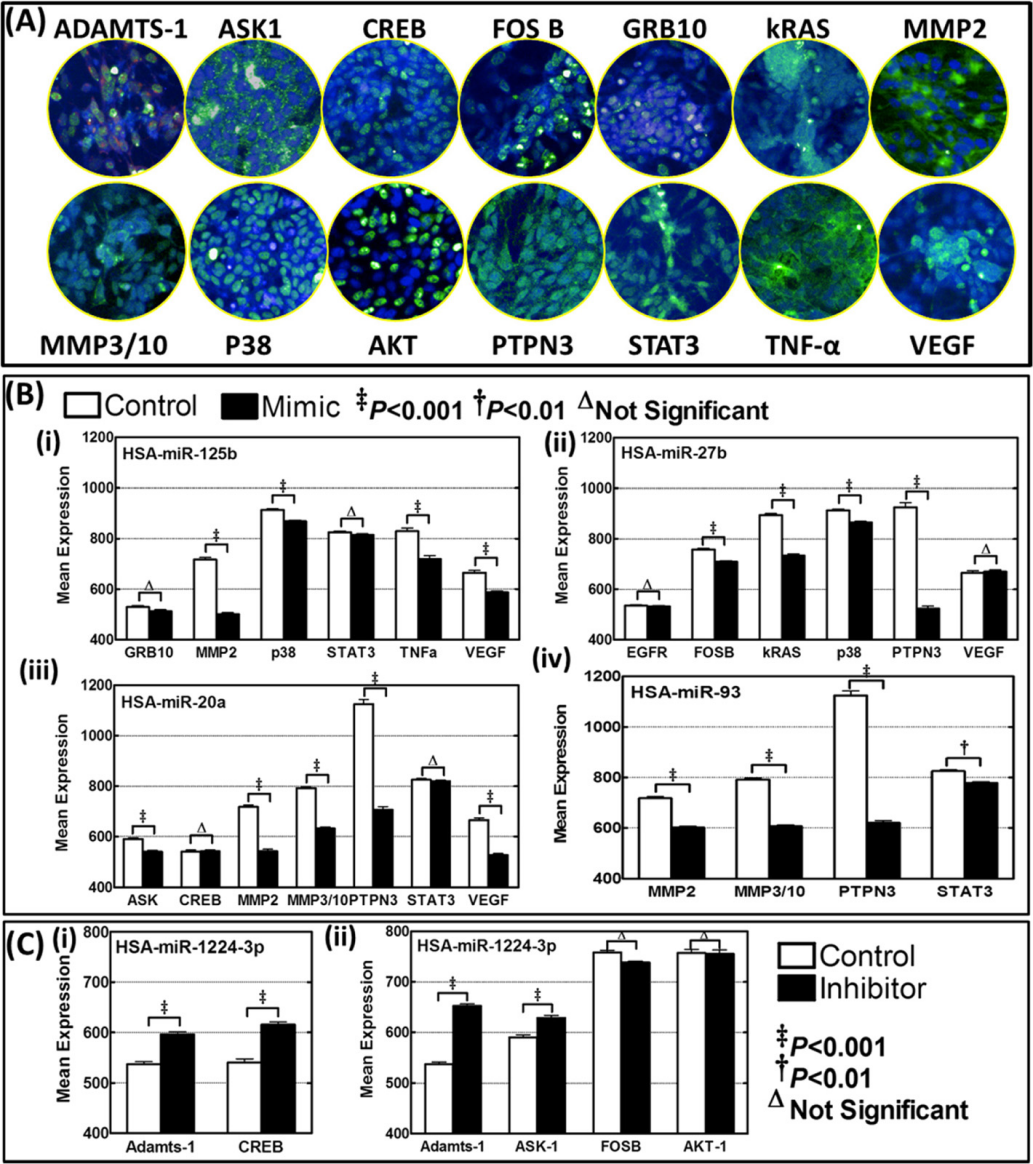
MetastamiRs dependent regulation of functional protein targets. **a** Operetta high-content confocal imaging showing immunofluorescence of ADAMTS-1, ASK-1, CREB, FOSB, GRB10, kRAS, MMP2, MMP3/10, p38, AKT, PTPN3, STAT3, TNFα and VEGFa in MSDACs. **b** Histograms of mean cell–Alexa Fluor intensity obtained from Columbus automated batch analysis showing alterations in the expression (*i*) GRB10, MMP2, p38, STAT3, TNFα and VEGF in cells with hsa-miR-125b mimic, (*ii*) EGFR FOSB, kRAS, p38, PTPN3 and VEGF in hsa-miR-27b mimic transfected cells, (*iii*) ASK1, CREB, MMP2, MMP3/10, PTPN3, STAT3and VEGF in MSDACs with hsa-miR-20a mimic and, (*iv*) MMP2, MMP3/10, PTPN3 and STAT3 with hsa-miR-93 mimic in MSDACs. **c** Histograms of mean cell–Alexa Fluor intensity showing alterations in the expression of (*i*) ADAMTS-1 and CREB with hsa-miR-1224-3p inhibition and, (*ii*) ADAMTS-1, ASK1, FOSB and AKT-1 with hsa-miR-1260 inhibition. Group-wise comparisons were performed with ANOVA with Tukey’s post-hoc correction

Further to define the direct role of the deregulated miRNAs of high-risk aggressive disease in the regulation of the target proteins that demonstrated profound alterations both in the metastatic tumors *in vivo* and in MSDACs *ex vivo* we examined the corresponding alterations of the target proteins after functionally mimicking or inhibiting miRNAs. First, MSDACs transiently transfected with mimics for hsa-miR-125b, hsa-miR-27b, hsa-miR-93 or hsa-miR-20a (those exhibited complete suppression in aggressive disease) and examined for the regulation of their corresponding target proteins (Fig. 6a). High-throughput quantitative confocal immunofluorescence demonstrated a significant (P < 0.001) inhibition of MMP2, p38, TNFα and VEGF in MSDACs in the presence of hsa-miR-125b mimic (Fig. 6b*i*). In addition, we observed a marginal decrease in GRB10 and STAT3 expression with hsa-miR-125b mimic. Similarly, functionally mimicking hsa-miR-27b resulted in the profound (P < 0.001) inhibition of FOSB, kRAS, p38 and PTPN3 (Fig. 6b*ii*). Interestingly, mimicking hsa-miR-27b did not inhibit the expression of EGFR and VEGF in MSDACs. On the other hand, MSDACs transfected with hsa-miR-20a exhibited significant (P < 0.001) inhibition of ASK1, MMP2, MMP3/10, PTPN3 and VEGF (Fig. 6b*iii*). We did not see any consistent inhibition of CREB and STAT3 at least with the mimic for hsa-miR-20a. Moreover, hsa-miR-93 mimic exhibited statistically significant inhibition of MMP2, MMP3/10, PTPN3 and STAT3 in this setting (Fig. 6b*iv*). Next, MSDACs transiently transfected with inhibitors for hsa-miR-1224-3p or hsa-miR-1260 (both showed profound induction in metastatic tumors) and examined for the alterations in protein targets. Inhibiting hsa-miR-1224-3p resulted in the significant (P < 0.001) induction of ADAMTS-1 and CREB (Fig. 6c*i*). Like-wise, inhibiting hsa-miR-1260 markedly (P < 0.001) induced ADAMTS-1 and ASK1 (Fig. 6c*ii*). However, inhibiting hsa-miR-1260 did not result in the induction of FOSB and AKT-1 in this setting. Together, these results clearly demonstrate the direct role of altered miRNAs observed in high-risk aggressive disease in the regulation of the protein targets identified in this setting and, thereby validates the translation of functional response of the neuroblastoma metastamiRs.

### Altered miRNAs serve as metastamiRs

To determine the relevance of metastamiRs in aggressive disease, we first clarified their role in cancer biological functions, network and communal molecular orchestrations, and tumor progression. For the miRNAs that were reorganized in high-risk NB, IPA “Core-Analysis” identified multiple networks classifying their roles in diseases including cancer and their specific functions (Additional file 1: Figure S1). Functionally, these miRNas were shown to be intrinsically involved in cell death and cell survival, inflammation, the cell cycle, cellular movement, DNA replication, recombination and repair, cell-to-cell signaling and interaction, and cellular growth and proliferation. These processes play defined roles in metastasis (Additional file 1: Figure S1). Convergence of all of the miRNA networks at the processes that endorse metastasis identify the altered miRNAs as metastamiRs. To further validate whether these miRNAs are recognized as metastamiRs in other tumor systems, we used the cancer miRNA profile database from mircancer.ecu.edu (release version of miRCancer June 2014), ingenuity pathway analysis of diseases and biological functions, and a manual literature survey.

Of the 74 metastamiRs, data mining in miRCancer identified at least 36 that were altered: hsa-let-7a, hsa-let-7b, hsa-let-7c, hsa-let-7e, hsa-let-7f, hsa-let-7 g, hsa-mir-1, hsa-mir-100, hsa-mir-122, hsa-mir-1250, hsa-mir-1258, hsa-mir-125b, hsa-mir-1280, hsa-mir-143, hsa-mir-148b, hsa-mir-149, hsa-mir-190b, hsa-mir-200b, hsa-mir-206, hsa-mir-20a, hsa-mir-220a, hsa-mir-23a, hsa-mir-27b, hsa-mir-29b-1, hsa-mir-30b, hsa-mir-30c-1, hsa-mir-32, hsa-mir-383, hsa-mir-432, hsa-mir-500, hsa-mir-601, hsa-mir-656, hsa-mir-765, hsa-mir-886-3p, hsa-mir-92a, and hsa-mir-93. Alterations in these metastamiRs were found in one or more of the following tumor systems: acute lymphoblastic leukemia, acute myeloid leukemia, acute promyelocytic leukemia, b-cell lymphoma, bladder cancer, breast cancer, bronchioloalveolar carcinoma, cervical cancer, cholangiocarcinoma, chronic lymphocytic leukemia, colon cancer, endometrial cancer, esophageal squamous cell carcinoma, follicular cancer, gastric cancer, glioblastoma, head and neck squamous cell carcinoma, hepatocellular carcinoma, laryngeal squamous cell carcinoma, liver cancer, lung cancer, malignant melanoma, mantle cell lymphoma, medulloblastoma, mesenchymal cancer, nasopharyngeal cancer, neuroblastoma, non-small cell lung cancer, oral squamous cell carcinoma, osteosarcoma, ovarian cancer, ovarian carcinoma, pancreatic cancer, papillary thyroid carcinoma, pituitary carcinoma, prostate cancer, renal cell carcinoma, thyroid cancer, and tongue cancer (data not shown).

Ingenuity pathway analysis, however, identified the expressional association of these metastamiRs in at least eighty different tumor systems (Additional file 2: Figure S2). Armed with the above information, we further explored the crucial role of metastamiRs in tumor cell metastasis. Additional file 5: Table S1 shows the list of identified miRNAs, their functions in the context of metastasis and tumor progression as per the published evidence, total number of their gene targets, and the references from which we obtained this information. Interestingly, almost all of the miRNAs identified in our high-risk metastatic neuroblastoma have been well characterized in terms of metastasis regulation (Additional file 5: Table S1), which could identify them as metastamiRs.

### Alterations in the targets of the identified metastamiRs are associated with poor clinical outcomes

To demonstrate the functional relevance of these metastamiRs to high-risk metastatic neuroblastoma and poor clinical outcomes, we examined the correlation of individual expression of their gene targets with overall survival (OS) in patients with neuroblastoma. Utilizing the gene expression data for a cohort of 88 human neuroblastoma patients, we examined the prognostic values for a total of 22 gene targets that had validated expression levels in our model. Kaplan-Meier plots showed a significant association between increased expression of ELK1, CDK4, MMP2, AURKβ, FRA, MYB, JUND, BIRC5, AKT2, SELE, TNFα (Additional file 3: Figure S3), NOS3, ESR1, KRTAP1-1, MMP3, MMP9, NF2, CXCR4, ADAMTS1, VEGF, CD54, EGFR, and AKT1 (Additional file 4: Figure 4) and poor OS in patients with neuroblastoma. This inverse association also reflects poor relapse-free survival in these patients (data not shown). Interestingly, there was a definite association between CREB1 loss and poor OS (Additional file 3: Figure 3). This CREB1 loss also resulted in poor relapse-free survival in children with neuroblastoma. Clinical outcome association analysis revealed a strong correlation between the increased expression of the genes listed above and stage progression, favorable → unfavorable disease and alive → died-of-disease (data not shown). Together, these data demonstrate the alterations of gene targets by the reorganization of metastamiRs in high-risk metastatic disease that drives poor clinical outcomes in children with neuroblastoma.

## Discussion

About 60 % of neuroblastoma patients with high-risk disease will relapse with hematogenous metastasis [6], despite intensive multimodal therapy [3, 5, 7–14]. Neuroblastoma is derived from embryonic neural crest cells that have a high potential to migrate. Since metastatic NB has a high mortality rate, understanding the molecular mechanism flow-through that is involved in neuroblastoma cell invasion and metastasis will help us to design more effective therapies against metastatic NB, particularly when we use appropriate clinically translatable animal models. I*n vitro* and *in vivo* approaches have identified numerous molecular markers that play crucial roles in the functional orchestration of neuroblastoma metastasis [19, 55–58]. After Ma and colleagues [32] demonstrated that miRNAs were the upstream regulators of the complex invasion-metastasis network in breast cancer, researchers began to uncover the roles of miRNA(s) in highly metastatic neuroblastoma [59, 60]. Transcriptome regulation occurs via miRNAs through multiple processes, including translational inhibition, destabilization, or RNA decay [33], all of which are considered important modulators of signal transduction pathways in metastatic progression.

Studies delineating the regulatory role of the miRNA in neuroblastoma metastasis have thus far been limited to the understanding of a single miRNA-associated target molecule’s dependent functions along the axis of metastasis [22, 43, 45, 46, 61]. Considering the complexity of the sequential steps involved in neuroblastoma metastasis, it is crucial to define the functional role of every miRNA (metastamiR) that plays a vital role in each step of metastasis. For the first time, utilizing a clinically translatable animal model of high-risk metastatic neuroblastoma, we have identified the cluster of metastamiRs, their functional translation in terms of target protein expression, and the association of such alterations with clinical outcomes.

In the current study, we used multiple complementary methodologies to identify neuroblastoma metastamiRs. First, this study employed a unique clinically translatable, reproducible mouse model of human high-risk metastatic neuroblastoma coupled with whole genome miRNA array technology. We screened differentially expressed miRNAs in metastatic tumors as opposed to non-metastatic tumors. We observed significant inter-animal variations in up-and downregulated miRNAs and under less stringent and more stringent conditions (see Fig. 1c). Examining this topic further, we validated the most distinctly deregulated over- and under—expressed miRNAs by RT-qPCR, utilized traverse analysis of altered miRNAs between the sample groups, characterized the reorganized miRNAs by compiling their interactions in ingenuity pathway analysis, expression in multiple tumor systems, and their functional role in metastasis and tumor progression. Further, we performed miRNA-target analysis, comprehensively identified the alterations in the expression and cellular localization of the target proteins in *ex vivo* and *in vivo* metastatic cells, recognized the direct influence of the altered miRNAs on the regulation of target proteins with mimic/inhibitor approach, and compiled the target proteins’ association with neuroblastoma patient outcomes.

Since the main objective was to identify the defined metastamiRs of neuroblastoma, we compared the traverse examination of four aggressive disease expression profiles to that of non-metastatic profiles to derive clear evidence of differential expression without equivocal outcomes. Interestingly, the outcome of the present work confirmed some findings from other neuroblastoma miRNA studies (http://mircancer.ecu.edu/browse.jsp) [59], but also essentially identified many new metastamiRs in this setting. Of the 74 metastamiRs identified in this study, we found an overlap of 16 metastamiRs, including Hsa-miR-148b, Hsa-miR-23a, Hsa-miR-100, Hsa-miR-93, Hsa-miR-125b, Hsa-miR-98, Hsa-miR-92a, Hsa-miR-29b, Hsa-miR-30c, Hsa-let-7a, Hsa-let-7b, Hsa-let-7c, Hsa-let-7e, Hsa-let-7f, and Hsa-let-7g, with the findings of other researchers. To our knowledge, this is the first such attempt to identify all-inclusive miRNAs that could play defined roles in metastasis, their functional translation, and their association with clinical outcomes, at least in the neuroblastoma setting. Almost all studies that investigated miRNA roles in neuroblastoma metastasis focused on understanding a single molecule. As discussed above, our approach to identify new metastamiRs in the neuroblastoma setting is directly related to the lack of any comprehensive approach studies for neuroblastoma or other tumor types. Limited overlap with similar studies in other cancers [59] may reflect the use of different detection platforms, miRBase releases, or tumor models or origins. In addition, results that are based solely on microarray data have a relatively high false-positive rate. Thus, we validated our microarray results with RT-qPCR for upregulation (Hsa-miR-1260; Hsa-miR-1224-3p) and downregulation (Hsa-miR-20a, Hsa-miR-27b, Hsa-miR-125b, Hsa-miR-93) profiles (see Fig. 3b).

Since it is important to characterize each of the reorganized miRNAs in neuroblastoma metastasis, and this activity was within the scope of this study, we extensively investigated the role of each miRNA in tumor progression and metastasis in multiple tumor systems, including neuroblastoma. Regulation of these miRNAs has been documented in many tumor systems and biological functions, including metastasis (Additional file 2: Figure S2). Though it is not practical to discuss each miRNA, we compiled their biological functions in the light of the published evidence that identifies them as metastamiRs (Additional file 5: Table S1). However, to understand their precise function in neuroblastoma metastasis and since expression profiles do not give us information about their biological functions, we measured the expressional alterations of key targets that were shown to play crucial roles in metastasis. For the first time, we quantified the expression and phosphorylation of 49 target proteins. Further, to capture the metastamiRs associated with translational alterations of the targets, we characterized their expression in metastatic cells *ex vivo* and in the manifold of aggressive metastatic tumors. The targets examined in the study are regulated by more than one metastamiR identified in this setting (see Table 1). Thus, to the best of our knowledge, the presented list of 49 deregulated metastasis-related protein targets of metastamiRs provides the most extensive list of neuroblastoma metastamiRs’ associated response. In this respect, the global approach in our study to assess the expression data in the manifold of metastatic tumors, all in the screening phase by microarrays, in the validation phase by RT-qPCR and response validation by target protein expressions and their miRNA dependent regulation, as mentioned above, proved to be advantageous. We also applied clinical outcome association analysis with the validated protein targets of the identified metastamiRs in the neuroblastoma setting. Induced expression (ELK1, CDK4, MMP2, AURKβ, FRA, MYB, JUND, BIRC5, AKT2, SELE, TNF-α, NOS3, ESR1, KRTAP1-1, MMP3, MMP9, NF2, CXCR4, ADAMTS1, VEGF, CD54, EGFR, and AKT1) and suppression (CREB1) of metastamiRs’ target proteins corresponded strongly with poor patient outcomes (Additional files 3: Figure S3 and Additional file 4: Figure S4).

## Conclusions

For the first time, this study of miRNAs profiles in non-metastatic and metastatic neuroblastoma identified a comprehensive list of 74 deregulated metastasis-associated miRNAs, termed metastamiRs. We primarily recognized new miRNAs associated with neuroblastoma metastasis, and also confirmed the results of other studies, as only a few miRNAs have been described as neuroblastoma metastamiRs. Further, our results demonstrated a significant modulation in the expression of 49 key metastasis-regulating protein targets of these metastamiRs in aggressive cells *ex vivo* and in the manifold of metastatic tumors *in vivo*. More importantly, the clinical outcome association analysis corresponded with poor overall and relapse-free survival. In conclusion, the current study identified a distinctive set of neuroblastoma metastamiRs that could serve as potential candidates for diagnostic markers for the switch from favorable to high-risk metastatic disease.

## Methods

### Cell culture

The SH-SY5Y human neuroblastoma cell line was obtained from ATCC (Manassas, VA) and was cultured and maintained as described earlier [54]. For passaging and for all experiments, the cells were detached using 0.25 % trypsin /1 % EDTA, re-suspended in complete medium, counted (Countess, Invitrogen), and incubated in a 95 % air/5 % CO_2_ humidified incubator.

### *In vivo* human neuroblastoma experiments—development of reproducible non-metastatic xenografts and mouse models of aggressive metastatic disease

All animal experiments conformed to American Physiological Society standards for animal care and were carried out in accordance with guidelines laid down by the National Research Council. All protocols were approved by the University of Oklahoma Health Sciences Center Institutional Animal Care and Use Committee and adhered to the ARRIVE guidelines. Seven-week-old athymic NCr-*nu/nu* nude mice (NCI, Frederick, MD) received SC injections of 5x10^6^ SH-SY5Y cells suspended in Matrigel (BD Biosciences) into their right flank. Animals were observed for xenograft and/or aggressive metastatic disease development for extended periods of time (Min 15; Max 60 days). Tumor growth, regression, and dissemination to distant sites were investigated by tumor volume measurements and non-invasive fluorescent imaging. For this purpose, we administered 2 nmol/100 μL of IntegriSense 750 (Perkin Elmer, Inc., Waltham, Inc.) intravenously through the tail vein. After 24 h, imaging was performed at excitation 745 and emission 800 using *In Vivo* Extreme (Carestream Health Inc., Rochester, NY). A reflectant image and x-ray CT image were also acquired to establish anatomical landmarks. Animals were euthanized by CO_2_ asphyxiation. The tumors from metastatic sites and non-metastatic xenografts were harvested and prepared as single-cell suspensions as described below. To reproduce high-risk aggressive disease, animals were injected with isolated and well-characterized clones of aggressive cells derived from individual metastatic sites, and observed for development of metastatic tumors. Parallel experiments were performed with cells derived and characterized from non-metastatic xenografts.

### Preparation of single-cell suspensions of tumor cells, cell sorting, and *ex vivo* maintenance

To derive tumor cells from individual metastatic sites and non-metastatic xenografts, we thoroughly minced pre-weighed tumor samples in cell dissociation buffer (DMEM media supplemented with 10 % FBS, penicillin/streptomycin, 200 U/mL Collagenase type IV, and 0.6 U/mL dispase). A 5 ml serological pipet of titrated cell suspension was allowed to incubate at 37 °C for 3 h for enzymatic dissociation, with mechanical dissociation every 15 min. At the end of the incubation, cells were filtered through a 70 μm filter and subjected to density centrifugation using Ficoll-Plaque Plus to separate viable cells from dead cells and tissue debris. Cells collected from the interface were diluted (1:3) in fresh DMEM:F12 with 10 % FBS and counted for viable cells using Countess. Metastatic site derived aggressive cells (MSDACs) were grown *ex vivo* in stem cell medium (DMEM:F12 with 1 % N2 Supplement, 2 % B27 Supplement, 20 ng/ml hPDGF, 100 ng/ml EGF, and 1 % antibiotic-antimycotic) at 37 °C, 5 % CO_2_. Suspensions were then sequentially characterized with karyotyping, whole genome array CGH analysis, *ex vivo* tumorosphere-forming capacity, and *in vivo* tumorigenicity.

### Total RNA/miRNA Isolation and whole genome miRNA microarray profiling

Total RNA/miRNA from parental SH-SY5Y, MSDACs, and a manifold of metastatic tumors was extracted using TRIzol reagent (Invitrogen) or miRNeasy kit (Qiagen), following the manufacturer’s protocol. RNA concentration and quality were determined using the RNA 6000 Nano assay on the Agilent 2100 Bioanalyzer (Agilent Technologies). miRNA expression profiles were performed using the human miRNA One Array V4 (Phalanx Biotech) that includes 1,884 unique miRNA probes and 144 experimental control probes. Each unique probe has 3 features. Probes contain 100 % of Sanger miRBase V18 miRNA content. Isolated miRNA (500 ng) was labeled with Cy3 using a Kreatech ULS miRNA labeling kit and hybridized using an miRNA One Array V4 kit (Phalanx) following the manufacturer’s protocol. Hybridized signals were detected by the microarray scanner G2600D (Agilent Technologies, Santa Clara, CA, USA) and quantified using ImageQuant (GE Healthcare Bio-Sciences, Pittsburgh, PA, USA). Complete matrix of the whole genome miRNA expression and their metadata are included in Additional files 6. Expression profiles of each feature were background-subtracted, normalized to internal U6 features, and compared between groups using Prism (GraphPad Software, Inc., La Jolla, CA, USA). To eliminate inter-animal variations that could equivocate expression outcomes within the group, we adopted non-metastatic control(s) → metastatic tumor(s) traverse analysis between every sample analyzed. For this, expression profile of background subtracted and array-normalized miRNAs of non-metastatic primary xenograft control-1 was first individually compared to the miRNA expression profiles of each aggressive metastatic tumors and, the overall and/or >2-fold up/down regulated miRNAs for each aggressive tumors are computed. The comparison analysis was then performed to identify miRNAs that were commonly up/down regulated (both overall as well as >2-fold change) across the aggressive tumors as opposed to control-1. Next a similar detailed approach was utilized to obtain the miRNAs that were commonly up/down regulated (both overall and >2-fold) across the aggressive tumors as opposed to non-metastatic primary xenograft control-2. Finally, crisscross data comparison analysis was performed between the data sets 1 and 2 to define the miRNAs that were up or down regulated in aggressive metastatic tumors, ruling out any intra-group variations associated equivocal outcomes. Commonly reorganized metastamiRs (upregulated and downregulated) across the manifold of metastatic tumors are further examined Ingenuity Pathway analysis, Target Scan, and miRBase to determine the gene targets.

### miRNA qPCR

In order to validate the miRNA expression obtained from whole genome profiling, expression of selected metastamiRs, including hsa-miR-1224-3p, hsa-miR-1260 (both significantly upregulated), hsa-miR-125b, hsa-miR-27b, hsa-miR-93 ,and hsa-miR-20a (all significantly downregulated) were confirmed using QPCR. Hsa-miR-U6 was used as an internal positive control. Briefly, poly (A) tailed (Poly (A) tailing kit, Life Technologies, Grand Island, NY, USA), miRNA was reverse transcribed using miRNA EasyScript™ cDNA synthesis kit (Applied Biological Materials Inc., Richmond, BC, Canada) as per the manufacturer’s protocol. QPCR sampling was performed in triplicate using an miRNA EasyScript™ cDNA Synthesis Kit (Applied Biological Materials, Inc.) following the manufacturer’s PCR conditions. U6 normalized expression was compared with the non-metastatic controls and expressed as a fold change. Group—wise comparisons were performed with two-way ANOVA with Tukey’s post-hoc correction (GraphPad Prism). Similarly, correlations in the expression patterns obtained from the whole genome profiling with the QPCR results were performed using GraphPad Prism.

### Functional characterization of metastamiRs and target analysis

To functionally characterize the identified miRNAs in tumor progression and metastasis we adopted three sequential approaches. First, utilizing Ingenuity Pathway Analysis (Ingenuity Systems, Inc.) we examined the credible intermolecular interactions and their association to cancer. Next, to underscore and identify metastamiRs’ relevance in tumor progression and to delineate neuroblastoma-specific shifts, we used the miRCancer (http://mircancer.ecu.edu) database, and compared the expression profiles in various human cancers, including neuroblastoma. Lastly, by extracting information from the published literature, we examined the defined functions of the miRNAs in the context of metastasis and tumor progression. Three inter-linked databases, ingenuity pathway analysis, miRBase, and microRNA.org, were used for targeted analysis of the identified metastamiRs. Extracted lists of targets were subsequently compared to each other and select targets that played crucial roles in metastasis and were regulated by more than one metastamiR were identified and examined for translational modulations.

### Immunoblotting

Total protein extraction and immunoblotting were performed as described earlier [62]. In MSDACs and metastatic tumors, we analyzed modulations in the altered expression of identified targets playing crucial roles in metastasis, including ADAMTS-1, pAKT1/2/3, AKT1/2/3, ASK1, AURKβ, Birc1, Birc2, Bric5, β-CATENIN, CASP8, CD54, CDK4, CREB, CTGF, CXCR4, CYCLIN-D1, EGFR, pEGFR, ELK1, ESR1, CFOS, FOSB, FRA, GRB10, pGSK3β, IL1α, JUND, kRAS, KRTAP1, MCP1, MEGF10, MMP2, MMP3, MMP9, MMP10, MTA2, MYB, cMYC, NF2, NOS3, pNOS3, P21, pP38, PTPN3, CLEAVED PARP, PKC, SDF-1β, SEMA3D, SELE, STAT3, TLR3, TNFα, TNFR1, and VEGF. Blots were stripped and reblotted with either anti-α-tubulin or anti-β-actin to determine equal loading of samples.

### Tissue microarray construction and quantitative immunohistochemistry

All mouse tissue microarray construction procedures were performed in the Stephenson Cancer Center-Cancer Tissue Pathology Core. For mouse neuroblastoma TMA, tumor tissues from non-metastatic xenograft-bearing animals and from multiple metastatic sites from high-risk aggressive disease-bearing animals were printed in duplicate. Immunohistochemical staining for GRB10, MYC, SEMA3D, KRTAP1-1, and MEGF10 was performed utilizing an automated Leica Bond III according to the manufacturer’s protocol using the Bond™ Polymer Refine detection system. Appropriate tissue morphologic/pathologic (H&E) controls and negative controls with no primary antibody (data not shown) were examined in parallel. The slides were micro-digitally scanned using an Aperio Scanscope (Aperio Technologies, Inc., Buffalo Grove, IL, USA) slide scanner. This allows the assembly of tissue collections in TMA with variable magnifications. We constructed virtual slides with digital histology. The digital images of the TMA were then analyzed for GRB10, MYC, SEMA3D, KRTAP1-1 or MEGF10 specific positivity using the Aperio TMALab™ software that is equipped with highly advanced algorithms for IHC and stain intensity including cytoplasmic-, nuclear-, membrane- and total- staining Intensity and/or counts quantification. Automated staining positivity and intensity was quantified in precisely located and identified (with grid/row/column coordinates) individual cores within the TMA using protein specific (GRB10, SEMA3D, KRTAP1-1-cytoplasmic; MYC-nuclear; MEGF10-membranous) image analysis algorithms. Core-specific metadata for the TMA was exported to excel and the group-wise comparisons were performed with two-way ANOVA with Tukey’s post-hoc correction (GraphPad Prism).

#### miRNA manipulations and quantitative high-throughput confocal immunofluorescence:

To define the effect of characterized metastamiRs on the putative target proteins, we adopted two approaches: (i) inhibited hsa-miR-1224-3p or hsa-miR-1260 (both significantly upregulated) and (ii) functionally mimicked hsa-miR-125b, hsa-miR-27b, hsa-miR-93 or hsa-miR-20a (all significantly downregulated) and examined for the miRNA-dependent modulations in protein targets. Transient transfection of MSDACs with hsa-miR-125b-, hsa-miR-27b-, hsa-miR-93- or hsa-miR-20a- mimics (MISSION® microRNA Mimics, Sigma-Aldrich) as well as hsa-miR-1224-3p- and hsa-miR-1260-inhibitors (MISSION® Synthetic miRNA Inhibitors, Sigma-Aldrich) were carried out by using either TurboFectin 8.0 reagent (Origene) or Neon electroporation transfection system (Life Technologies). We examined the cellular localization and expression levels of ADAMTS-1, ASK-1, CREB, FOSB, GRB10, EGFR, kRAS, MMP2, MMP3/10, p38, AKT, PTPN3, STAT3, TNFα and VEGFa in MSDACs using Operetta high content quantitative confocal imaging. Paraformaldehyde fixed cells were permeabilized (0.25 % Triton X-100), blocked, and labelled with corresponding antibody. They were then tagged with Alexa Fluor 488 conjugated anti-mouse or anti-rabblit secondary antibody (Abcam). The nucleus was counter-labeled with DAPI. At least sixty-three fields/well with a minimum of 3Z planes were analyzed with integrated Columbus image analysis software. Unbiased automated batch analysis was performed and the cells-total number, mean/well cell–Alexa Fluor intensity were computed. Group-wise comparisons were performed with ANOVA with Tukey’s post-hoc correction (GraphPad Prism).

### Clinical outcome association analysis

We used the R2: microarray analysis and visualization platform (http://r2.amc.nl) created by Dr. Jan Koster at the Academic Medical Center (AMC), Amsterdam, to examine the association of the metastamiRs’ key targets, i.e., those that showed defined alterations in protein expression/phosphorylation with immunoblotting, with overall survival of patients with neuroblastoma. This web-based application correlates a select gene expression profile with clinical outcomes using samples from various cohorts of patients, and permitted us to demonstrate the significance of altered genes in high-risk disease and their relevance to clinical outcomes. For this study, we utilized a cohort of 88 untreated primary human neuroblastoma samples and examined the prognostic values for ELK1, CDK4, CREB1, MMP2, AURKβ, FRA, MYB, JUND, BIRC5, AKT2, SELE, TNFα, NOS3, ESR1, KRTAP1-1, MMP3, NF2, CXCR4, ADAMTS1, CD54, EGFR, and AKT1 expression. Available clinical information on these patients include age group (<=18 m – 48; >18 m – 40), gender (Male 53, Female 35), INSS (Stage 1 – 8, Stage 2 – 15, Stage 3 – 13, Stage 4 – 40, Stage 4S – 12), MYCN status (amplified 16, non-amplified 72), recurrence/progression (yes 35, not detected 53), alive (yes, 55, no 33) etc., (http://r2.amc.nl). The association of each gene’s expression with the overall survival probability was plotted in Kaplan-Meier plots constructed for a follow-up of 240 months.

## Additional files

Additional file 1: Figure S1. Ingenuity interaction networks for the miRNAs reorganized in aggressive high-risk metastatic neuroblastoma. All networks identified by IPA converge at biological functions that endorses metastatsis and, thereby signify their role as metastamiRs. Additional file 2: Figure S2. Ingenuity pathway analysis: Heat map showing the association of the identified metastamiRs in multiple tumor systems. Box sizes correspond to the number of focus molecules and the color intensity is the –log(p-value). Numerical labels in the boxes corresponds to the tumor type. Additional file 3: Figure S3. Kaplan Meier plots showing clinical outcomes in a cohort of 88 neuroblastoma patients in association with the expression pattern of metastamiRs’ protein targets, ELK1, CDK4, CREB1, MMP2, AURKβ, FRA, MYB, JUND, BIRC5, AKT2, SELE, TNFα. All these targets showed induced expression levels (except CREB1) in MSDACs (compared to parental SH-SY5Y) and manifold of metastatic tumors (compared with non-metastatic xenograft) as examined with immunoblotting. Gene expression-associated clinical outcomes were assessed using the web-based R2: microarray analysis and visualization platform. Induced expression of ELK1, CDK4, MMP2, AURKβ, FRA, MYB, JUND, BIRC5, AKT2, SELE and TNFα as well as decreased expression of CREB1 corresponded to the poor overall survival in NB patients. Additional file 4: Figure S4. Kaplan Meier plots showing clinical outcomes in a cohort of 88 neuroblastoma patients in association with the expression pattern of metastamiRs’ targets NOS3, ESR1, SELE, KRTAP1-1, MMP3, NF2, ELK-1, CXCR4, ADAMTS-1, ICAM-1, EGFR and ATK-1. All these targets showed induced expression levels in MSDACs (compared to parental SH-SY5Y) and manifold of metastatic tumors (compared with non-metastatic xenograft) as examined with immunoblotting. Additional file 5: Table S1. List of reorganized miRNAs (52 up regulated and 22 down regulated), their defined (published evidence) functions in the context of metastasis and tumor progression, corresponding number of gene targets and the miRNA→metastatsis citation index. Almost all of the miRNAs identified in high-risk metastatic neuroblastomas has been well characterized in the perspective of metastasis regulation and, could be classified as metastamiRs. Additional file 6:
**Reorganization of metastamiRs in the evolution of metastatic aggressive neuroblastoma cells.**

## Abbreviations

NB: Neuroblastoma
OS: Overall survival
EMT: Epithelial-mesenchymal transition
CSCs: Cancer stem cells
MSDACs: Metastatic sites derived aggressive cells
TMA: Tissue microarray
