# Supplementary material for: Reorganization of metastamiRs in the evolution of metastatic aggressive neuroblastoma cells
Source: BMC Genomics. 2015 Jul 7;16(1):501. doi: 10.1186/s12864-015-1642-x (PMC4491873; doi:10.1186/s12864-015-1642-x)
Supplement: Additional file 1: Figure S1. — Ingenuity interaction networks for the miRNAs reorganized in aggressive high-risk metastatic neuroblastoma. All networks identified by IPA converge at biological functions that endorses metastatsis and, thereby signify their role as metastamiRs. [file 12864_2015_1642_MOESM1_ESM.pptx]

## Slide 1
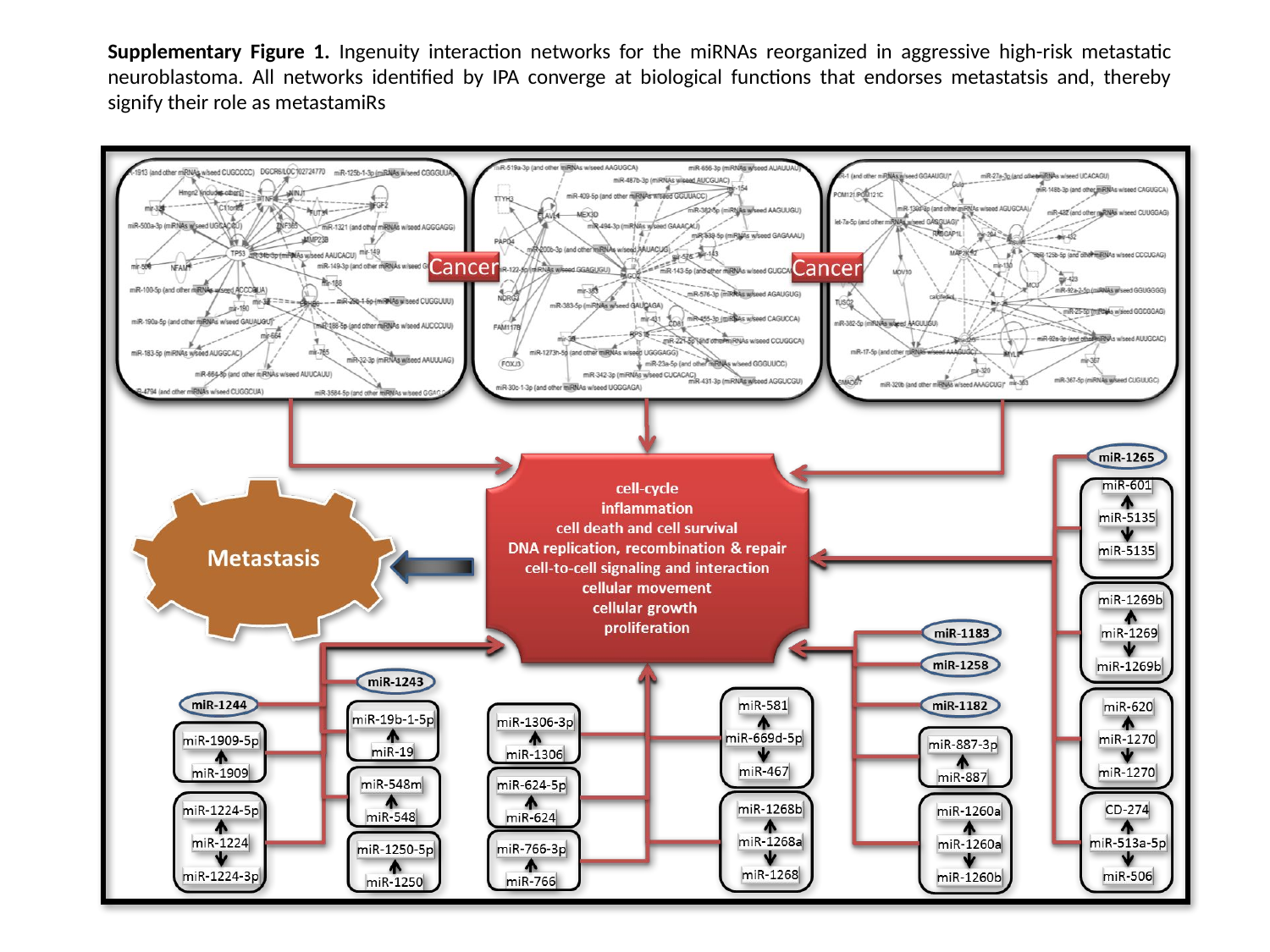

Supplementary Figure 1. Ingenuity interaction networks for the miRNAs reorganized in aggressive high-risk metastatic neuroblastoma. All networks identified by IPA converge at biological functions that endorses metastatsis and, thereby signify their role as metastamiRs
