# Supplementary material for: Reorganization of metastamiRs in the evolution of metastatic aggressive neuroblastoma cells
Source: BMC Genomics. 2015 Jul 7;16(1):501. doi: 10.1186/s12864-015-1642-x (PMC4491873; doi:10.1186/s12864-015-1642-x)
Supplement: Additional file 2: Figure S2. — Ingenuity pathway analysis: Heat map showing the association of the identified metastamiRs in multiple tumor systems. Box sizes correspond to the number of focus molecules and the color intensity is the –log(p-value). Numerical labels in the boxes corresponds to the tumor type. [file 12864_2015_1642_MOESM2_ESM.pptx]

## Slide 1
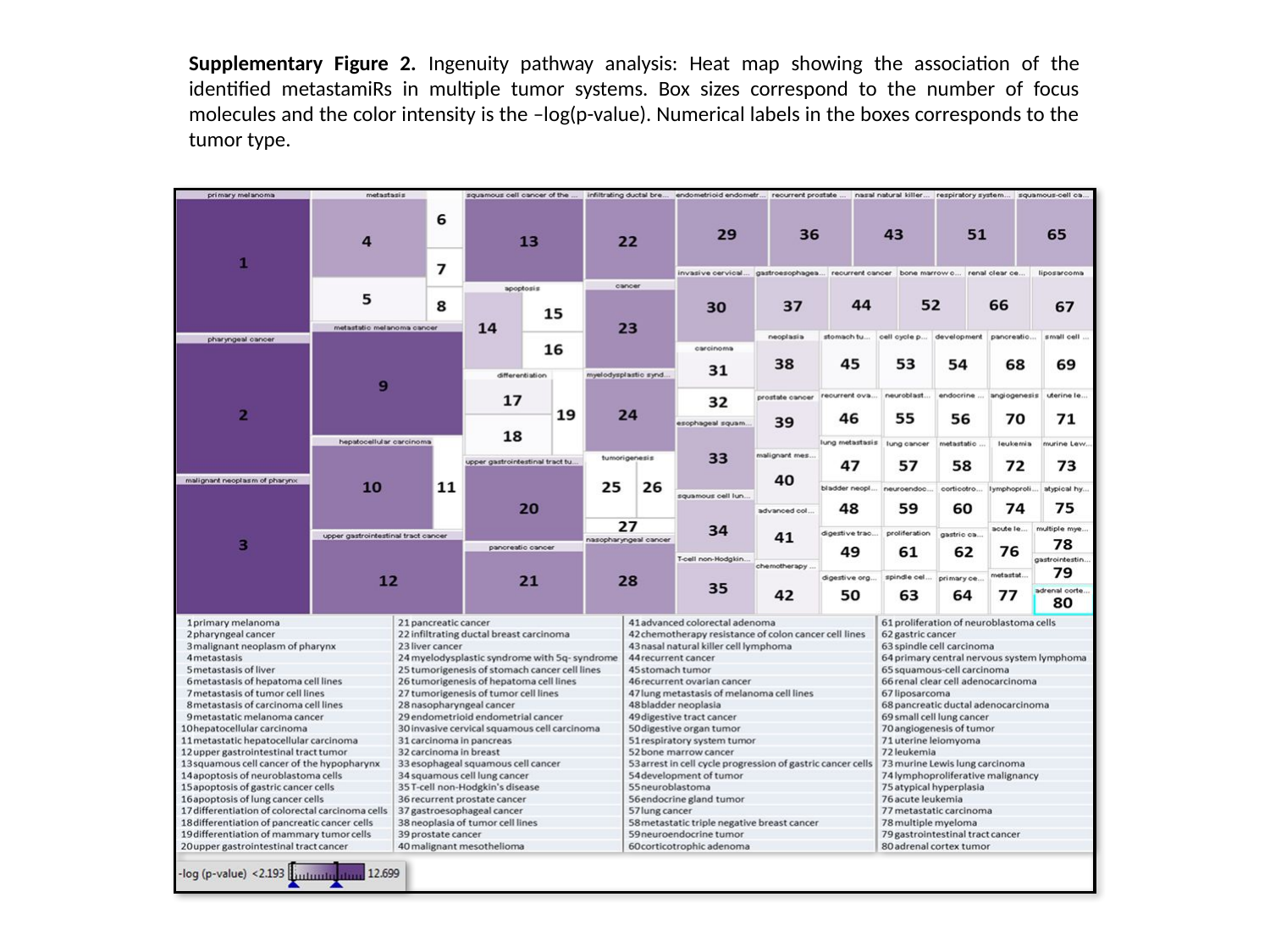

Supplementary Figure 2. Ingenuity pathway analysis: Heat map showing the association of the identified metastamiRs in multiple tumor systems. Box sizes correspond to the number of focus molecules and the color intensity is the –log(p-value). Numerical labels in the boxes corresponds to the tumor type.
