# Supplementary material for: Reorganization of metastamiRs in the evolution of metastatic aggressive neuroblastoma cells
Source: BMC Genomics. 2015 Jul 7;16(1):501. doi: 10.1186/s12864-015-1642-x (PMC4491873; doi:10.1186/s12864-015-1642-x)
Supplement: Additional file 3: Figure S3. — Kaplan Meier plots showing clinical outcomes in a cohort of 88 neuroblastoma patients in association with the expression pattern of metastamiRs’ protein targets, ELK1, CDK4, CREB1, MMP2, AURKβ, FRA, MYB, JUND, BIRC5, AKT2, SELE, TNFα. All these targets showed induced expression levels (except CREB1) in MSDACs (compared to parental SH-SY5Y) and manifold of metastatic tumors (compared with non-metastatic xenograft) as examined with immunoblotting. Gene expression-associated clinical outcomes were assessed using the web-based R2: microarray analysis and visualization platform. Induced expression of ELK1, CDK4, MMP2, AURKβ, FRA, MYB, JUND, BIRC5, AKT2, SELE and TNFα as well as decreased expression of CREB1 corresponded to the poor overall survival in NB patients. [file 12864_2015_1642_MOESM3_ESM.pptx]

## Slide 1
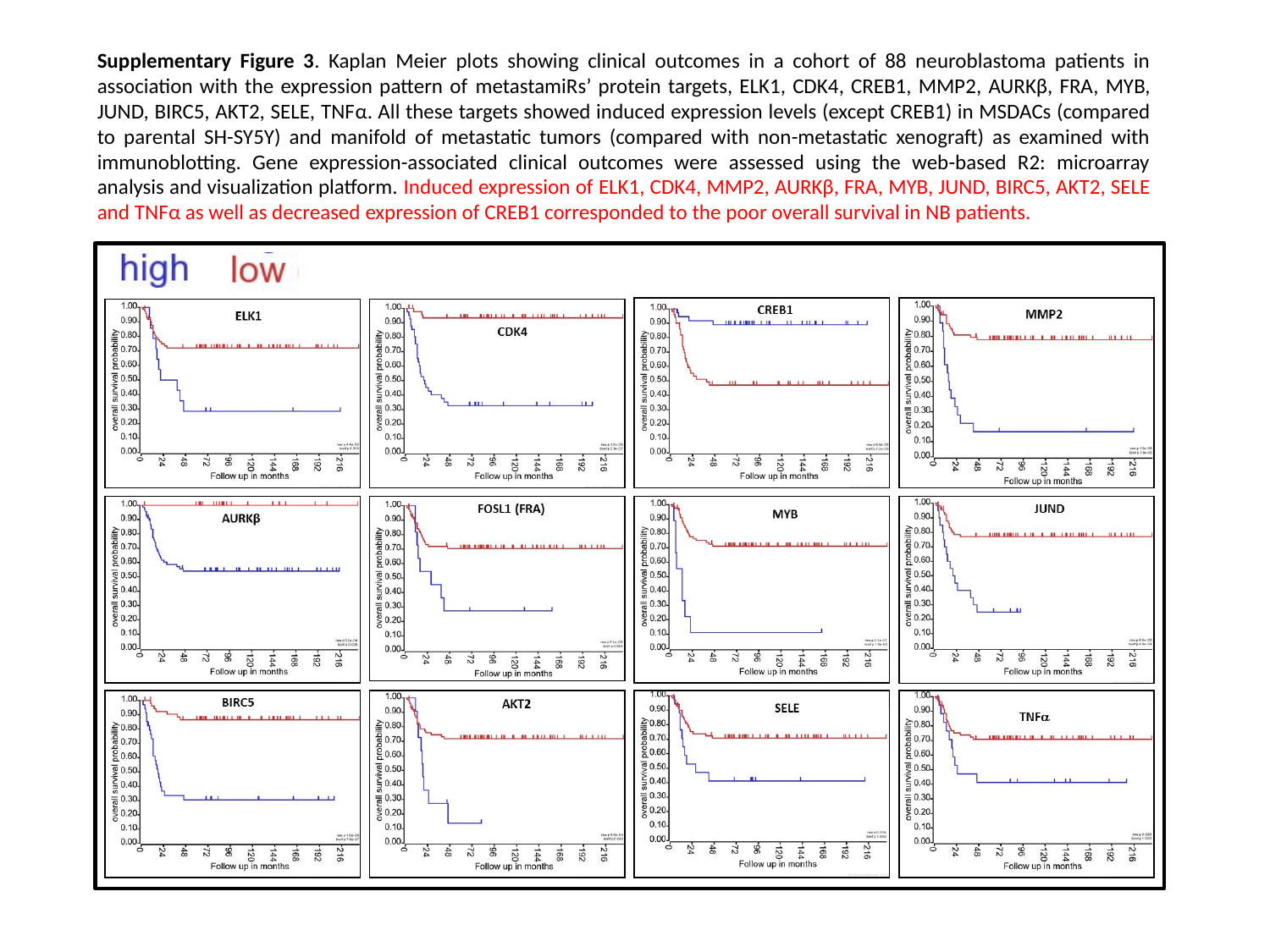

Supplementary Figure 3. Kaplan Meier plots showing clinical outcomes in a cohort of 88 neuroblastoma patients in association with the expression pattern of metastamiRs’ protein targets, ELK1, CDK4, CREB1, MMP2, AURKβ, FRA, MYB, JUND, BIRC5, AKT2, SELE, TNFα. All these targets showed induced expression levels (except CREB1) in MSDACs (compared to parental SH-SY5Y) and manifold of metastatic tumors (compared with non-metastatic xenograft) as examined with immunoblotting. Gene expression-associated clinical outcomes were assessed using the web-based R2: microarray analysis and visualization platform. Induced expression of ELK1, CDK4, MMP2, AURKβ, FRA, MYB, JUND, BIRC5, AKT2, SELE and TNFα as well as decreased expression of CREB1 corresponded to the poor overall survival in NB patients.
