# Supplementary material for: Reorganization of metastamiRs in the evolution of metastatic aggressive neuroblastoma cells
Source: BMC Genomics. 2015 Jul 7;16(1):501. doi: 10.1186/s12864-015-1642-x (PMC4491873; doi:10.1186/s12864-015-1642-x)
Supplement: Additional file 4: Figure S4. — Kaplan Meier plots showing clinical outcomes in a cohort of 88 neuroblastoma patients in association with the expression pattern of metastamiRs’ targets NOS3, ESR1, SELE, KRTAP1-1, MMP3, NF2, ELK-1, CXCR4, ADAMTS-1, ICAM-1, EGFR and ATK-1. All these targets showed induced expression levels in MSDACs (compared to parental SH-SY5Y) and manifold of metastatic tumors (compared with non-metastatic xenograft) as examined with immunoblotting. [file 12864_2015_1642_MOESM4_ESM.pptx]

## Slide 1
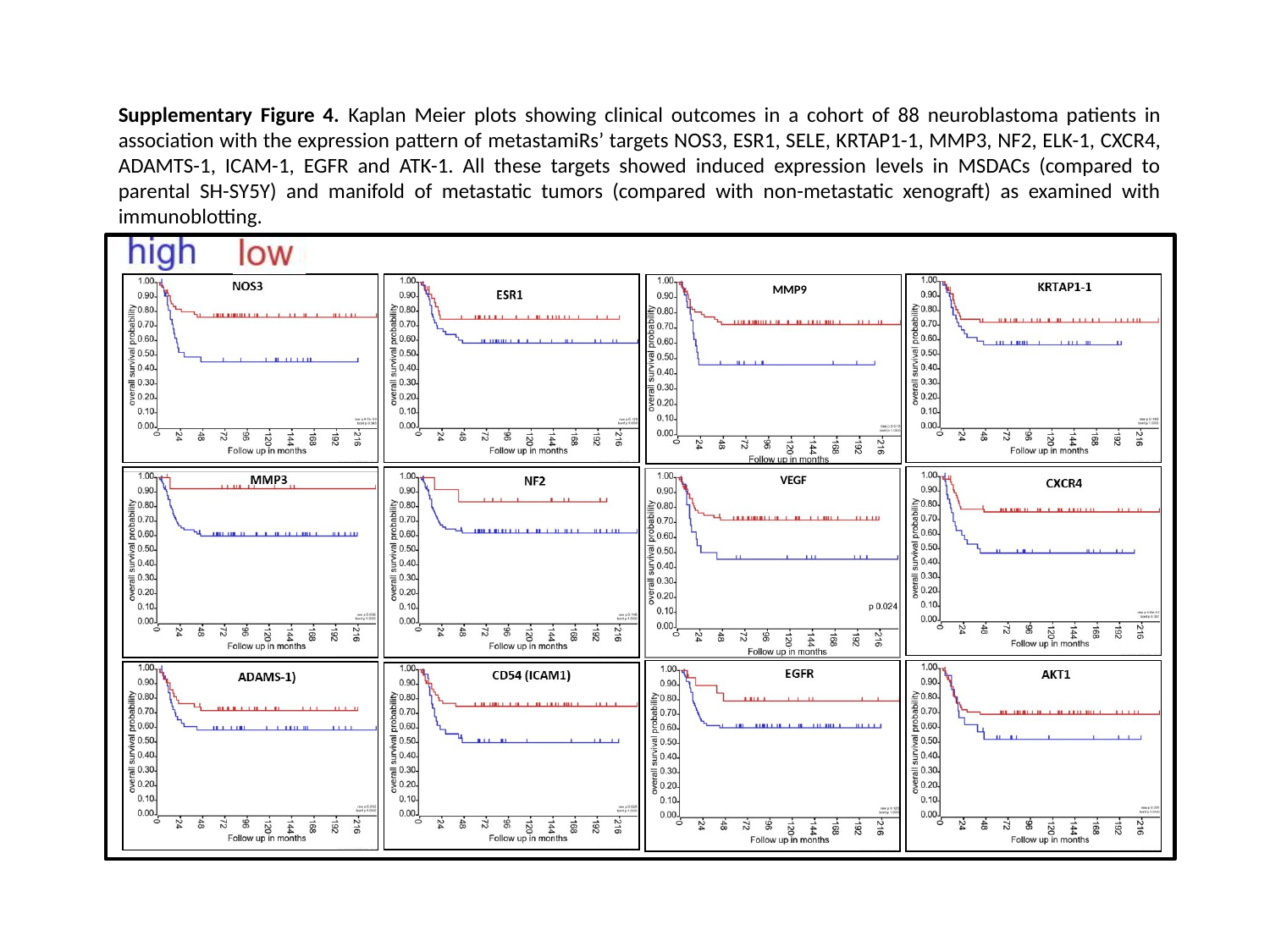

Supplementary Figure 4. Kaplan Meier plots showing clinical outcomes in a cohort of 88 neuroblastoma patients in association with the expression pattern of metastamiRs’ targets NOS3, ESR1, SELE, KRTAP1-1, MMP3, NF2, ELK-1, CXCR4, ADAMTS-1, ICAM-1, EGFR and ATK-1. All these targets showed induced expression levels in MSDACs (compared to parental SH-SY5Y) and manifold of metastatic tumors (compared with non-metastatic xenograft) as examined with immunoblotting.
MMP9
VEGF
