# Supplementary material for: Reorganization of metastamiRs in the evolution of metastatic aggressive neuroblastoma cells
Source: BMC Genomics. 2015 Jul 7;16(1):501. doi: 10.1186/s12864-015-1642-x (PMC4491873; doi:10.1186/s12864-015-1642-x)
Supplement: Additional file 5: Table S1. — List of reorganized miRNAs (52 up regulated and 22 down regulated), their defined (published evidence) functions in the context of metastasis and tumor progression, corresponding number of gene targets and the miRNA→metastatsis citation index. Almost all of the miRNAs identified in high-risk metastatic neuroblastomas has been well characterized in the perspective of metastasis regulation and, could be classified as metastamiRs. [file 12864_2015_1642_MOESM5_ESM.docx]

**Supplementary Table 1.** List of reorganized miRNAs (52 up regulated and 22 down regulated), their defined (published evidence) functions in the context of metastasis and tumor progression, corresponding number of gene targets and the miRNA→metastatsis citation index. Almost all of the miRNAs identified in high-risk metastatic neuroblastomas has been well characterized in the perspective of metastasis regulation and, could be classified as metastamiRs.

| **miRNA** | **Experimentally observed Functions** | **Targets** | **References** |
| --- | --- | --- | --- |
| **hsa-let-7a** | Tumor suppressor, Inhibit Invasion and metastasis, Prognostic marker | 20 | ([1-5](#_ENREF_1)) |
| **hsa-miR-1** | Tumor suppressor, Inhibit Invasion and metastasis, Prognostic marker | 1165 | ([6-14](#_ENREF_6)) |
| **hsa-miR-1182** | Diagnostic Biomarker | 629 | ([15](#_ENREF_15)) |
| **hsa-miR-1183** | Tumor suppressor, Inhibit Invasion and metastasis | 403* | ([16](#_ENREF_16)) |
| **hsa-miR-122** | Induces MET, tumor suppressor, Inhibit Invasion and metastasis, | 11 | ([17-23](#_ENREF_17)) |
| **hsa-miR-1224-3p** | Cancer Predictive marker | 1292 | ([24](#_ENREF_24)) |
| **hsa-miR-1224-5p** | Cancer (chemo-refractory metastatic) prognosis Predictive marker | 595 | ([25-27](#_ENREF_25)) |
| **hsa-miR-1243** | Cancer Invasiveness | 479 | ([28](#_ENREF_28)) |
| **hsa-miR-1244** | Cancer Progression | 351 | ([29](#_ENREF_29)) |
| **hsa-miR-1250** | Cancer progression (in cancer stem cells) | 308 | ([30](#_ENREF_30)) |
| **hsa-miR-1258** | Tumor suppressor, Inhibit Invasion and metastasis | 385 | ([31-33](#_ENREF_31)) |
| **hsa-miR-125b-1*** | Induce cellular senescence and tumor suppressor, inhibit metastasis | 26 | ([34](#_ENREF_34), [35](#_ENREF_35)) |
| **hsa-miR-1260** | Tumor suppressor | 1347 | ([36](#_ENREF_36)) |
| **hsa-miR-1265** | Predictive marker | 440 | ([37](#_ENREF_37)) |
| **hsa-miR-1268** | Cancer Progression | 673 | ([38](#_ENREF_38)) |
| **hsa-miR-1280** | Inhibit Invasion and metastasis | 162 | ([39](#_ENREF_39)) |
| **hsa-miR-1306** | Cancer Progression | 218 | ([40](#_ENREF_40)) |
| **hsa-miR-1308** | Cancer diagnostic biomarker | 1299 | ([41](#_ENREF_41), [42](#_ENREF_42)) |
| **hsa-miR-1321** | Inhibit tumor suppressor | 1611 | ([43](#_ENREF_43)) |
| **hsa-miR-149*** | Inhibit metastasis, suppress integrin signaling | 1225 | ([44](#_ENREF_44), [45](#_ENREF_45)) |
| **hsa-miR-188-5p** | Predictive marker | 426 | ([26](#_ENREF_26), [46](#_ENREF_46)) |
| **hsa-miR-190** | Inhibit angiogenesis and metastasis | 413 | ([47](#_ENREF_47)) |
| **hsa-miR-200b** | Inhibit EMT, invasion and metastasis, promote tumor suppressor | 1236 | ([48-65](#_ENREF_48)) |
| **hsa-miR-206** | Tumor suppressor, Inhibit migration, invasion, metastasis and angiogenesis | 1165 | ([5](#_ENREF_5), [50](#_ENREF_50), [66-74](#_ENREF_66)) |
| **hsa-miR-23a*** | Promote metastasis | 1505 | ([75-78](#_ENREF_75)) |
| **hsa-miR-29b-1*** | Tumor suppressor | 13 | ([79](#_ENREF_79)) |
| **hsa-miR-30b*** | Tumor suppressor, Inhibit Invasion, metastasis and angiogenesis, Prognostic marker | 1594 | ([80-83](#_ENREF_80)) |
| **hsa-miR-30c-1*** | Invasion and metastasis | 3 | ([84-87](#_ENREF_84)) |
| **hsa-miR-32*** | Anti-Invasion, anti-Metastasis | 1138 | ([88](#_ENREF_88), [89](#_ENREF_89)) |
| **hsa-miR-320b** | Anti-Invasion , Anti-Metastasis, Anti-angiogenesis | 1075 | ([90](#_ENREF_90), [91](#_ENREF_91)) |
| **hsa-miR-382** | Prognostic maker | 449 | ([92](#_ENREF_92)) |
| **hsa-miR-383** | Promote cell proliferation, invasion and metastasis, Prognostic maker | 541 | ([93-100](#_ENREF_93)) |
| **hsa-miR-409-5p** | Promote EMT, tumorigenesis | 315 | ([101](#_ENREF_101), [102](#_ENREF_102)) |
| **hsa-miR-432** | Promote progression and metastasis, Predictive biomarker | 492 | ([103-109](#_ENREF_103)) |
| **hsa-miR-513a-5p** | Promote tumor progression and metastasis, Prognostic maker | 831 | ([110-112](#_ENREF_110)) |
| **hsa-miR-548m** | Prognostic maker | 399 | ([113](#_ENREF_113)) |
| **hsa-miR-576-3p** | Promote cell proliferation and apoptosis | 401 | ([114](#_ENREF_114)) |
| **hsa-miR-601** | Tumor suppressor | 381 | ([59](#_ENREF_59), [115](#_ENREF_115)) |
| **hsa-miR-608** | Promote tumor progression and metastasis, Prognostic and predctive maker | 2208 | ([116](#_ENREF_116)) |
| **hsa-miR-620** | Tumor suppressor | 543 | ([117](#_ENREF_117)) |
| **hsa-miR-664*** | Inhibit tumor suppressor, Prognostic maker | 241 | ([118-120](#_ENREF_118)) |
| **hsa-miR-765** | Inhibit Invasion and metastasis, Prognostic maker | 1464 | ([26](#_ENREF_26), [121-125](#_ENREF_121)) |
| **hsa-miR-766** | Tumor suppressor, Inhibit Invasion and metastasis, Predictive marker | 1097 | ([126-129](#_ENREF_126)) |
| **hsa-miR-92a-2*** | Promote tumorigenesis and metastasis | 13 | ([130](#_ENREF_130)) |
| **hsa-miR-100** | Inhibit migration, invasion, EMT and stemness | 193 | ([131-137](#_ENREF_131)) |
| **hsa-miR-125b** | Anti-Metastasis, tumor suppressor | 1534 | ([34](#_ENREF_34), [35](#_ENREF_35), [138-142](#_ENREF_138)) |
| **hsa-miR-1269** | Cancer Progression | 549 | ([40](#_ENREF_40)) |
| **hsa-miR-143*** | Inhibits cell migration and invasion | 771 | ([143-152](#_ENREF_143)) |
| **hsa-miR-148b** | Tumor suppressor | 1198 | ([153](#_ENREF_153), [154](#_ENREF_154)) |
| **hsa-miR-1909*** | Predictive marker | 1782 | ([26](#_ENREF_26)) |
| **hsa-miR-19b-1*** | Promote cell cycle, cell migration or invasion, and repress senescence and apoptosis | 12 | ([155-157](#_ENREF_155)) |
| **hsa-miR-220a** | Tumor suppressor | 1418 | ([158](#_ENREF_158)) |
| **hsa-miR-27b** | Inhibit tumor progression and angiogenesis | 1651 | ([159-161](#_ENREF_159)) |
| **hsa-miR-32** | Inhibits the proliferation and invasion | 1138 | ([88](#_ENREF_88), [89](#_ENREF_89), [162](#_ENREF_162)) |
| **hsa-miR-431*** | Induce Tumorigenesis | 573 | ([163](#_ENREF_163)) |
| **hsa-miR-500*** | Inhibit invasion, Diagnostic markers | 323 | ([164](#_ENREF_164), [165](#_ENREF_165)) |
| **hsa-miR-519c-3p** | Anti-angiogenesis | 705 | ([166](#_ENREF_166))* |
| **hsa-miR-581** | Tumor suppressor | 370 | ([167](#_ENREF_167)) |
| **hsa-miR-593** | Tumor suppressor | 628 | ([115](#_ENREF_115))* |
| **hsa-miR-624*** | Cancer diagnosis | 551 | ([168](#_ENREF_168), [169](#_ENREF_169)) |
| **hsa-miR-656** | Inhibit Tumorigenesis | 72 | ([170-172](#_ENREF_170)) |
| **hsa-miR-887** | Prognosis and predictive biomarker | 192 | ([173](#_ENREF_173), [174](#_ENREF_174)) |
| **hsa-miR-93** | Enhances angiogenesis and metastasis | 1418 | ([175-177](#_ENREF_175)) |

**References:**

1. Long XB*, et al.* (2009) Let-7a microRNA functions as a potential tumor suppressor in human laryngeal cancer. *Oncol Rep* 22(5):1189-1195.

2. Markou A, Yousef GM, Stathopoulos E, Georgoulias V, & Lianidou E (2014) Prognostic significance of metastasis-related microRNAs in early breast cancer patients with a long follow-up. *Clin Chem* 60(1):197-205.

3. McCarty MF (2012) Metformin may antagonize Lin28 and/or Lin28B activity, thereby boosting let-7 levels and antagonizing cancer progression. *Med Hypotheses* 78(2):262-269.

4. Namwat N*, et al.* (2012) Expression profiles of oncomir miR-21 and tumor suppressor let-7a in the progression of opisthorchiasis-associated cholangiocarcinoma. *Asian Pacific journal of cancer prevention : APJCP* 13 Suppl:65-69.

5. Vickers MM*, et al.* (2012) Stage-dependent differential expression of microRNAs in colorectal cancer: potential role as markers of metastatic disease. *Clinical & experimental metastasis* 29(2):123-132.

6. Xu L*, et al.* (2014) Tumor suppressor miR-1 restrains epithelial-mesenchymal transition and metastasis of colorectal carcinoma via the MAPK and PI3K/AKT pathway. *Journal of translational medicine* 12(1):244.

7. Han C, Yu Z, Duan Z, & Kan Q (2014) Role of microRNA-1 in human cancer and its therapeutic potentials. *BioMed research international* 2014:428371.

8. Zhao Q*, et al.* (2014) Correlation between the expression levels of miR-1 and PIK3CA in non-small-cell lung cancer and their relationship with clinical characteristics and prognosis. *Future Oncol* 10(1):49-57.

9. Huang D*, et al.* (2014) miRNA27a is a biomarker for predicting chemosensitivity and prognosis in metastatic or recurrent gastric cancer. *J Cell Biochem* 115(3):549-556.

10. Fu HL*, et al.* (2013) Altered miRNA expression is associated with differentiation, invasion, and metastasis of esophageal squamous cell carcinoma (ESCC) in patients from Huaian, China. *Cell biochemistry and biophysics* 67(2):657-668.

11. Li Z, Gu X, Fang Y, Xiang J, & Chen Z (2012) microRNA expression profiles in human colorectal cancers with brain metastases. *Oncology letters* 3(2):346-350.

12. Hudson RS*, et al.* (2012) MicroRNA-1 is a candidate tumor suppressor and prognostic marker in human prostate cancer. *Nucleic Acids Res* 40(8):3689-3703.

13. Beltran AS*, et al.* (2011) Suppression of breast tumor growth and metastasis by an engineered transcription factor. *PLoS One* 6(9):e24595.

14. Clarke MR, Baker EE, Weyant RJ, Hill L, & Carty SE (1997) Proliferative Activity in Pancreatic Endocrine Tumors: Association with Function, Metastases, and Survival. *Endocrine pathology* 8(3):181-187.

15. Zhang T*, et al.* (2013) MicroRNA-1322 regulates ECRG2 allele specifically and acts as a potential biomarker in patients with esophageal squamous cell carcinoma. *Mol Carcinog* 52(8):581-590.

16. Hu M*, et al.* (2012) Functional polymorphism in the EpCAM gene is associated with occurrence and advanced disease status of cervical cancer in Chinese population. *Molecular biology reports* 39(7):7303-7309.

17. Chen CL*, et al.* (2014) Baculovirus-Mediated miRNA Regulation to Suppress Hepatocellular Carcinoma Tumorigenicity and Metastasis. *Molecular therapy : the journal of the American Society of Gene Therapy*.

18. Wang SC*, et al.* (2014) MicroRNA-122 triggers mesenchymal-epithelial transition and suppresses hepatocellular carcinoma cell motility and invasion by targeting RhoA. *PLoS One* 9(7):e101330.

19. Chen Q*, et al.* (2014) Plasma miR-122 and miR-192 as potential novel biomarkers for the early detection of distant metastasis of gastric cancer. *Oncol Rep* 31(4):1863-1870.

20. Zhang H*, et al.* (2013) Circulating microRNAs in relation to EGFR status and survival of lung adenocarcinoma in female non-smokers. *PLoS One* 8(11):e81408.

21. Iino I*, et al.* (2013) Effect of miR-122 and its target gene cationic amino acid transporter 1 on colorectal liver metastasis. *Cancer Sci* 104(5):624-630.

22. Coulouarn C, Factor VM, Andersen JB, Durkin ME, & Thorgeirsson SS (2009) Loss of miR-122 expression in liver cancer correlates with suppression of the hepatic phenotype and gain of metastatic properties. *Oncogene* 28(40):3526-3536.

23. Tsai WC*, et al.* (2009) MicroRNA-122, a tumor suppressor microRNA that regulates intrahepatic metastasis of hepatocellular carcinoma. *Hepatology* 49(5):1571-1582.

24. Miah S*, et al.* (2012) An evaluation of urinary microRNA reveals a high sensitivity for bladder cancer. *Br J Cancer* 107(1):123-128.

25. Mosakhani N*, et al.* (2012) MicroRNA profiling predicts survival in anti-EGFR treated chemorefractory metastatic colorectal cancer patients with wild-type KRAS and BRAF. *Cancer genetics* 205(11):545-551.

26. Della Vittoria Scarpati G*, et al.* (2012) A specific miRNA signature correlates with complete pathological response to neoadjuvant chemoradiotherapy in locally advanced rectal cancer. *Int J Radiat Oncol Biol Phys* 83(4):1113-1119.

27. Nymark P*, et al.* (2011) Integrative analysis of microRNA, mRNA and aCGH data reveals asbestos- and histology-related changes in lung cancer. *Genes Chromosomes Cancer* 50(8):585-597.

28. Peng Y*, et al.* (2014) Expression profile and clinical significance of microRNAs in papillary thyroid carcinoma. *Molecules* 19(8):11586-11599.

29. Ottman R, Nguyen C, Lorch R, & Chakrabarti R (2014) MicroRNA expressions associated with progression of prostate cancer cells to antiandrogen therapy resistance. *Mol Cancer* 13:1.

30. Chang CJ*, et al.* (2011) p53 regulates epithelial-mesenchymal transition and stem cell properties through modulating miRNAs. *Nat Cell Biol* 13(3):317-323.

31. Tang D*, et al.* (2013) The expression and clinical significance of microRNA-1258 and heparanase in human breast cancer. *Clinical biochemistry* 46(10-11):926-932.

32. Liu H, Chen X, Gao W, & Jiang G (2012) The expression of heparanase and microRNA-1258 in human non-small cell lung cancer. *Tumour biology : the journal of the International Society for Oncodevelopmental Biology and Medicine* 33(5):1327-1334.

33. Zhang L, Sullivan PS, Goodman JC, Gunaratne PH, & Marchetti D (2011) MicroRNA-1258 suppresses breast cancer brain metastasis by targeting heparanase. *Cancer Res* 71(3):645-654.

34. Nyholm AM*, et al.* (2014) miR-125b induces cellular senescence in malignant melanoma. *BMC dermatology* 14:8.

35. Sun YM, Lin KY, & Chen YQ (2013) Diverse functions of miR-125 family in different cell contexts. *Journal of hematology & oncology* 6:6.

36. Ma XQ, Wang LP, Luo QC, & Cai JC (2013) [Relationship between the expression level of miR-29c and biological behavior of gastric cancer]. *Zhonghua Zhong Liu Za Zhi* 35(5):325-330.

37. Xu CZ*, et al.* (2013) Gene and microRNA expression reveals sensitivity to paclitaxel in laryngeal cancer cell line. *International journal of clinical and experimental pathology* 6(7):1351-1361.

38. Ali S, Almhanna K, Chen W, Philip PA, & Sarkar FH (2010) Differentially expressed miRNAs in the plasma may provide a molecular signature for aggressive pancreatic cancer. *Am J Transl Res* 3(1):28-47.

39. Majid S*, et al.* (2012) MicroRNA-1280 inhibits invasion and metastasis by targeting ROCK1 in bladder cancer. *PLoS One* 7(10):e46743.

40. Watahiki A*, et al.* (2011) MicroRNAs associated with metastatic prostate cancer. *PLoS One* 6(9):e24950.

41. Jones CI*, et al.* (2012) Identification of circulating microRNAs as diagnostic biomarkers for use in multiple myeloma. *Br J Cancer* 107(12):1987-1996.

42. Jaiswal R*, et al.* (2012) Microparticle conferred microRNA profiles--implications in the transfer and dominance of cancer traits. *Mol Cancer* 11:37.

43. Liu F*, et al.* (2013) Identification of aberrant microRNA expression pattern in pediatric gliomas by microarray. *Diagnostic pathology* 8:158.

44. Perez-Rivas LG*, et al.* (2014) A microRNA signature associated with early recurrence in breast cancer. *PLoS One* 9(3):e91884.

45. Chan SH*, et al.* (2014) MicroRNA-149 targets GIT1 to suppress integrin signaling and breast cancer metastasis. *Oncogene* 33(36):4496-4507.

46. Hu J*, et al.* (2014) Human miR-1228 as a stable endogenous control for the quantification of circulating microRNAs in cancer patients. *Int J Cancer* 135(5):1187-1194.

47. Hao Y*, et al.* (2014) The synergistic regulation of VEGF-mediated angiogenesis through miR-190 and target genes. *Rna* 20(8):1328-1336.

48. Humphries B*, et al.* (2014) MicroRNA-200b targets protein kinase Calpha and suppresses triple-negative breast cancer metastasis. *Carcinogenesis*.

49. Cristobal I*, et al.* (2014) Deregulation of miR-200b, miR-200c and miR-429 indicates its potential relevant role in patients with colorectal cancer liver metastasis. *J Surg Oncol* 110(4):484-485.

50. Wang B, Li J, Sun M, Sun L, & Zhang X (2014) miRNA expression in breast cancer varies with lymph node metastasis and other clinicopathologic features. *IUBMB life* 66(5):371-377.

51. Wang Z, Humphries B, Xiao H, Jiang Y, & Yang C (2014) MicroRNA-200b suppresses arsenic-transformed cell migration by targeting protein kinase Calpha and Wnt5b-protein kinase Calpha positive feedback loop and inhibiting Rac1 activation. *J Biol Chem* 289(26):18373-18386.

52. Williams LV, Veliceasa D, Vinokour E, & Volpert OV (2013) miR-200b inhibits prostate cancer EMT, growth and metastasis. *PLoS One* 8(12):e83991.

53. Peng F*, et al.* (2013) Direct targeting of SUZ12/ROCK2 by miR-200b/c inhibits cholangiocarcinoma tumourigenesis and metastasis. *Br J Cancer* 109(12):3092-3104.

54. Zhang HF*, et al.* (2014) miR-200b suppresses invasiveness and modulates the cytoskeletal and adhesive machinery in esophageal squamous cell carcinoma cells via targeting Kindlin-2. *Carcinogenesis* 35(2):292-301.

55. Tang H*, et al.* (2013) miR-200b and miR-200c as prognostic factors and mediators of gastric cancer cell progression. *Clin Cancer Res* 19(20):5602-5612.

56. Toiyama Y*, et al.* (2014) Serum miR-200c is a novel prognostic and metastasis-predictive biomarker in patients with colorectal cancer. *Ann Surg* 259(4):735-743.

57. Sun M*, et al.* (2014) RKIP and HMGA2 regulate breast tumor survival and metastasis through lysyl oxidase and syndecan-2. *Oncogene* 33(27):3528-3537.

58. Peng B*, et al.* (2013) MicroRNA-200b targets CREB1 and suppresses cell growth in human malignant glioma. *Mol Cell Biochem* 379(1-2):51-58.

59. Scheffer AR*, et al.* (2014) Circulating microRNAs in serum: novel biomarkers for patients with bladder cancer? *World journal of urology* 32(2):353-358.

60. Madhavan D*, et al.* (2012) Circulating miRNAs as surrogate markers for circulating tumor cells and prognostic markers in metastatic breast cancer. *Clin Cancer Res* 18(21):5972-5982.

61. Au SL*, et al.* (2012) Enhancer of zeste homolog 2 epigenetically silences multiple tumor suppressor microRNAs to promote liver cancer metastasis. *Hepatology* 56(2):622-631.

62. Kurashige J*, et al.* (2012) MicroRNA-200b regulates cell proliferation, invasion, and migration by directly targeting ZEB2 in gastric carcinoma. *Annals of surgical oncology* 19 Suppl 3:S656-664.

63. Ding W*, et al.* (2012) miR-200b restoration and DNA methyltransferase inhibitor block lung metastasis of mesenchymal-phenotype hepatocellular carcinoma. *Oncogenesis* 1:e15.

64. Baffa R*, et al.* (2009) MicroRNA expression profiling of human metastatic cancers identifies cancer gene targets. *The Journal of pathology* 219(2):214-221.

65. Mees ST*, et al.* (2010) EP300--a miRNA-regulated metastasis suppressor gene in ductal adenocarcinomas of the pancreas. *Int J Cancer* 126(1):114-124.

66. Wang J*, et al.* (2014) miR-206 inhibits cell migration through direct targeting of the actin-binding protein Coronin 1C in triple-negative breast cancer. *Molecular oncology*.

67. Ren J*, et al.* (2014) MicroRNA-206 suppresses gastric cancer cell growth and metastasis. *Cell & bioscience* 4:26.

68. Fu Y, Jiang BQ, Wu Y, Li ZD, & Zhuang ZG (2013) [Hsa-miR-206 inhibits the migration and invasion of breast cancer by targeting Cx43]. *Zhonghua Yi Xue Za Zhi* 93(36):2890-2894.

69. Vimalraj S, Miranda PJ, Ramyakrishna B, & Selvamurugan N (2013) Regulation of breast cancer and bone metastasis by microRNAs. *Dis Markers* 35(5):369-387.

70. Bao YP*, et al.* (2013) Roles of microRNA-206 in osteosarcoma pathogenesis and progression. *Asian Pacific journal of cancer prevention : APJCP* 14(6):3751-3755.

71. Zhang T*, et al.* (2011) Down-regulation of MiR-206 promotes proliferation and invasion of laryngeal cancer by regulating VEGF expression. *Anticancer Res* 31(11):3859-3863.

72. Leite KR*, et al.* (2013) MicroRNA expression profiles in the progression of prostate cancer--from high-grade prostate intraepithelial neoplasia to metastasis. *Urologic oncology* 31(6):796-801.

73. Wang X, Ling C, Bai Y, & Zhao J (2011) MicroRNA-206 is associated with invasion and metastasis of lung cancer. *Anatomical record* 294(1):88-92.

74. Negrini M & Calin GA (2008) Breast cancer metastasis: a microRNA story. *Breast Cancer Res* 10(2):203.

75. Cheng L*, et al.* (2014) MicroRNA-23a promotes neuroblastoma cell metastasis by targeting CDH1. *Oncology letters* 7(3):839-845.

76. Li X*, et al.* (2013) c-MYC-regulated miR-23a/24-2/27a cluster promotes mammary carcinoma cell invasion and hepatic metastasis by targeting Sprouty2. *J Biol Chem* 288(25):18121-18133.

77. Wang Z, Wei W, & Sarkar FH (2012) miR-23a, a critical regulator of "migR"ation and metastasis in colorectal cancer. *Cancer discovery* 2(6):489-491.

78. Tang HL*, et al.* (2012) [Expression and clinical significance of miR-23a and metastasis suppressor 1 in colon carcinoma]. *Zhonghua bing li xue za zhi Chinese journal of pathology* 41(1):28-32.

79. Zhou H, Wang K, Hu Z, & Wen J (2013) TGF-beta1 alters microRNA profile in human gastric cancer cells. *Chinese journal of cancer research = Chung-kuo yen cheng yen chiu* 25(1):102-111.

80. Zhao H, Xu Z, Qin H, Gao Z, & Gao L (2014) miR-30b regulates migration and invasion of human colorectal cancer via SIX1. *Biochem J* 460(1):117-125.

81. Shao C*, et al.* (2012) Amplification and up-regulation of microRNA-30b in oral squamous cell cancers. *Arch Oral Biol* 57(8):1012-1017.

82. Hafez MM*, et al.* (2012) MicroRNAs and metastasis-related gene expression in Egyptian breast cancer patients. *Asian Pacific journal of cancer prevention : APJCP* 13(2):591-598.

83. Gaziel-Sovran A*, et al.* (2011) miR-30b/30d regulation of GalNAc transferases enhances invasion and immunosuppression during metastasis. *Cancer Cell* 20(1):104-118.

84. Dobson JR*, et al.* (2014) hsa-mir-30c promotes the invasive phenotype of metastatic breast cancer cells by targeting NOV/CCN3. *Cancer cell international* 14:73.

85. Heinzelmann J*, et al.* (2014) MicroRNAs with prognostic potential for metastasis in clear cell renal cell carcinoma: a comparison of primary tumors and distant metastases. *Annals of surgical oncology* 21(3):1046-1054.

86. Bockhorn J*, et al.* (2013) MicroRNA-30c targets cytoskeleton genes involved in breast cancer cell invasion. *Breast Cancer Res Treat* 137(2):373-382.

87. Mu YP & Su XL (2012) Polymorphism in pre-miR-30c contributes to gastric cancer risk in a Chinese population. *Med Oncol* 29(3):1723-1732.

88. Zhang J*, et al.* (2014) microRNA-32 inhibits the proliferation and invasion of the SGC-7901 gastric cancer cell line. *Oncology letters* 7(1):270-274.

89. Wu W*, et al.* (2013) The relationship between and clinical significance of MicroRNA-32 and phosphatase and tensin homologue expression in colorectal cancer. *Genes Chromosomes Cancer* 52(12):1133-1140.

90. Wu YY*, et al.* (2014) miR-320 regulates tumor angiogenesis driven by vascular endothelial cells in oral cancer by silencing neuropilin 1. *Angiogenesis* 17(1):247-260.

91. Yao J*, et al.* (2012) GNAI1 Suppresses Tumor Cell Migration and Invasion and is Post-Transcriptionally Regulated by Mir-320a/c/d in Hepatocellular Carcinoma. *Cancer biology & medicine* 9(4):234-241.

92. Sarver AL*, et al.* (2013) MicroRNAs at the human 14q32 locus have prognostic significance in osteosarcoma. *Orphanet journal of rare diseases* 8:7.

93. Vilming Elgaaen B*, et al.* (2014) Global miRNA expression analysis of serous and clear cell ovarian carcinomas identifies differentially expressed miRNAs including miR-200c-3p as a prognostic marker. *BMC Cancer* 14:80.

94. Lee CH*, et al.* (2013) MicroRNA-Regulated Protein-Protein Interaction Networks and Their Functions in Breast Cancer. *International journal of molecular sciences* 14(6):11560-11606.

95. He Z*, et al.* (2013) Downregulation of miR-383 promotes glioma cell invasion by targeting insulin-like growth factor 1 receptor. *Med Oncol* 30(2):557.

96. Li KK*, et al.* (2013) MiR-383 is downregulated in medulloblastoma and targets peroxiredoxin 3 (PRDX3). *Brain pathology* 23(4):413-425.

97. Wang XM*, et al.* (2012) [MicroRNA383 regulates expression of PRDX3 in human medulloblastomas]. *Zhonghua bing li xue za zhi Chinese journal of pathology* 41(8):547-552.

98. Costa FF*, et al.* (2011) Identification of microRNAs as potential prognostic markers in ependymoma. *PLoS One* 6(10):e25114.

99. Boguslawska J, Wojcicka A, Piekielko-Witkowska A, Master A, & Nauman A (2011) MiR-224 targets the 3'UTR of type 1 5'-iodothyronine deiodinase possibly contributing to tissue hypothyroidism in renal cancer. *PLoS One* 6(9):e24541.

100. Lian J*, et al.* (2010) Downregulation of microRNA-383 is associated with male infertility and promotes testicular embryonal carcinoma cell proliferation by targeting IRF1. *Cell death & disease* 1:e94.

101. Josson S*, et al.* (2014) Stromal fibroblast-derived miR-409 promotes epithelial-to-mesenchymal transition and prostate tumorigenesis. *Oncogene* 0.

102. Josson S*, et al.* (2014) miR-409-3p/-5p Promotes Tumorigenesis, Epithelial-to-Mesenchymal Transition, and Bone Metastasis of Human Prostate Cancer. *Clin Cancer Res* 20(17):4636-4646.

103. Sun L*, et al.* (2013) MicroRNA expression profiles of circulating microvesicles in hepatocellular carcinoma. *Acta gastro-enterologica Belgica* 76(4):386-392.

104. Kim YW*, et al.* (2014) Differential microRNA expression signatures and cell type-specific association with Taxol resistance in ovarian cancer cells. *Drug design, development and therapy* 8:293-314.

105. Yao T*, et al.* (2013) Exploration of tumor-suppressive microRNAs silenced by DNA hypermethylation in cervical cancer. *Virology journal* 10:175.

106. Nemlich Y*, et al.* (2013) MicroRNA-mediated loss of ADAR1 in metastatic melanoma promotes tumor growth. *J Clin Invest* 123(6):2703-2718.

107. Huang YH*, et al.* (2012) Identification of postoperative prognostic microRNA predictors in hepatocellular carcinoma. *PLoS One* 7(5):e37188.

108. D'Angelo D*, et al.* (2012) Altered microRNA expression profile in human pituitary GH adenomas: down-regulation of miRNA targeting HMGA1, HMGA2, and E2F1. *J Clin Endocrinol Metab* 97(7):E1128-1138.

109. Chen YX*, et al.* (2012) [Differential expression analysis of prolactinoma-related microRNAs]. *Zhonghua Yi Xue Za Zhi* 92(5):320-323.

110. Mosakhani N*, et al.* (2013) MicroRNA expression profiles in metastatic and non-metastatic giant cell tumor of bone. *Histology and histopathology* 28(5):671-678.

111. Pentheroudakis G*, et al.* (2013) Global microRNA profiling in favorable prognosis subgroups of cancer of unknown primary (CUP) demonstrates no significant expression differences with metastases of matched known primary tumors. *Clinical & experimental metastasis* 30(4):431-439.

112. Wu L, Chen Z, Zhang J, & Xing Y (2012) Effect of miR-513a-5p on etoposide-stimulating B7-H1 expression in retinoblastoma cells. *Journal of Huazhong University of Science and Technology. Medical sciences = Hua zhong ke ji da xue xue bao. Yi xue Ying De wen ban = Huazhong keji daxue xuebao. Yixue Yingdewen ban* 32(4):601-606.

113. Lwin T*, et al.* (2013) A microenvironment-mediated c-Myc/miR-548m/HDAC6 amplification loop in non-Hodgkin B cell lymphomas. *J Clin Invest* 123(11):4612-4626.

114. Coskun E*, et al.* (2013) MicroRNA profiling reveals aberrant microRNA expression in adult ETP-ALL and functional studies implicate a role for miR-222 in acute leukemia. *Leuk Res* 37(6):647-656.

115. Nygren MK*, et al.* (2014) Identifying microRNAs regulating B7-H3 in breast cancer: the clinical impact of microRNA-29c. *Br J Cancer* 110(8):2072-2080.

116. Tokarz P & Blasiak J (2012) The role of microRNA in metastatic colorectal cancer and its significance in cancer prognosis and treatment. *Acta biochimica Polonica* 59(4):467-474.

117. Zhao Z*, et al.* (2014) GPC5, a tumor suppressor, is regulated by miR-620 in lung adenocarcinoma. *Molecular medicine reports* 9(6):2540-2546.

118. Bueno RC*, et al.* (2014) ATM down-regulation is associated with poor prognosis in sporadic breast carcinomas. *Annals of oncology : official journal of the European Society for Medical Oncology / ESMO* 25(1):69-75.

119. Wang Z*, et al.* (2013) Upregulation of miR-2861 and miR-451 expression in papillary thyroid carcinoma with lymph node metastasis. *Med Oncol* 30(2):577.

120. Yang H*, et al.* (2013) MicroRNAs regulate methionine adenosyltransferase 1A expression in hepatocellular carcinoma. *J Clin Invest* 123(1):285-298.

121. Leung YK*, et al.* (2014) Hsa-miRNA-765 as a key mediator for inhibiting growth, migration and invasion in fulvestrant-treated prostate cancer. *PLoS One* 9(5):e98037.

122. Papp G, Krausz T, Stricker TP, Szendroi M, & Sapi Z (2014) SMARCB1 expression in epithelioid sarcoma is regulated by miR-206, miR-381, and miR-671-5p on Both mRNA and protein levels. *Genes Chromosomes Cancer* 53(2):168-176.

123. Dai N*, et al.* (2013) Alteration of the microRNA expression profile in human osteosarcoma cells transfected with APE1 siRNA. *Neoplasma* 60(4):384-394.

124. Guidi M*, et al.* (2010) Overexpression of miR-128 specifically inhibits the truncated isoform of NTRK3 and upregulates BCL2 in SH-SY5Y neuroblastoma cells. *BMC molecular biology* 11:95.

125. Tombol Z*, et al.* (2010) MicroRNA expression profiling in benign (sporadic and hereditary) and recurring adrenal pheochromocytomas. *Modern pathology : an official journal of the United States and Canadian Academy of Pathology, Inc* 23(12):1583-1595.

126. Li X, Shi Y, Yin Z, Xue X, & Zhou B (2014) An eight-miRNA signature as a potential biomarker for predicting survival in lung adenocarcinoma. *Journal of translational medicine* 12:159.

127. Liang H*, et al.* (2013) MicroRNAs contribute to promyelocyte apoptosis in As2O3-treated APL cells. *Cell Physiol Biochem* 32(6):1818-1829.

128. Sand M*, et al.* (2012) Microarray analysis of microRNA expression in cutaneous squamous cell carcinoma. *Journal of dermatological science* 68(3):119-126.

129. Hummel R*, et al.* (2011) Chemotherapy-induced modification of microRNA expression in esophageal cancer. *Oncol Rep* 26(4):1011-1017.

130. Li M*, et al.* (2014) miR-92a family and their target genes in tumorigenesis and metastasis. *Exp Cell Res* 323(1):1-6.

131. Zhang N*, et al.* (2014) MicroRNA-100 promotes migration and invasion through mammalian target of rapamycin in esophageal squamous cell carcinoma. *Oncol Rep* 32(4):1409-1418.

132. Wang M*, et al.* (2014) Loss of miR-100 enhances migration, invasion, epithelial-mesenchymal transition and stemness properties in prostate cancer cells through targeting Argonaute 2. *Int J Oncol* 45(1):362-372.

133. Chen P, Zhao X, & Ma L (2013) Downregulation of microRNA-100 correlates with tumor progression and poor prognosis in hepatocellular carcinoma. *Mol Cell Biochem* 383(1-2):49-58.

134. Huang JS, Egger ME, Grizzle WE, & McNally LR (2013) MicroRNA-100 regulates IGF1-receptor expression in metastatic pancreatic cancer cells. *Biotechnic & histochemistry : official publication of the Biological Stain Commission* 88(7):397-402.

135. Xu C*, et al.* (2013) miRNA-100 inhibits human bladder urothelial carcinogenesis by directly targeting mTOR. *Mol Cancer Ther* 12(2):207-219.

136. Liu J*, et al.* (2012) MicroRNA-100 is a potential molecular marker of non-small cell lung cancer and functions as a tumor suppressor by targeting polo-like kinase 1. *BMC Cancer* 12:519.

137. Peng DX, Luo M, Qiu LW, He YL, & Wang XF (2012) Prognostic implications of microRNA-100 and its functional roles in human epithelial ovarian cancer. *Oncol Rep* 27(4):1238-1244.

138. Ferracin M*, et al.* (2013) miR-125b targets erythropoietin and its receptor and their expression correlates with metastatic potential and ERBB2/HER2 expression. *Mol Cancer* 12(1):130.

139. Wang HJ*, et al.* (2013) miR-125b regulates side population in breast cancer and confers a chemoresistant phenotype. *J Cell Biochem* 114(10):2248-2257.

140. Tang F*, et al.* (2012) MicroRNA-125b induces metastasis by targeting STARD13 in MCF-7 and MDA-MB-231 breast cancer cells. *PLoS One* 7(5):e35435.

141. Zhang Y*, et al.* (2011) miR-125b is methylated and functions as a tumor suppressor by regulating the ETS1 proto-oncogene in human invasive breast cancer. *Cancer Res* 71(10):3552-3562.

142. Glud M*, et al.* (2010) Downregulation of miR-125b in metastatic cutaneous malignant melanoma. *Melanoma Res* 20(6):479-484.

143. Xia H*, et al.* (2014) miR-143 inhibits NSCLC cell growth and metastasis by targeting Limk1. *International journal of molecular sciences* 15(7):11973-11983.

144. Chen Y, Ma C, Zhang W, Chen Z, & Ma L (2014) Down regulation of miR-143 is related with tumor size, lymph node metastasis and HPV16 infection in cervical squamous cancer. *Diagnostic pathology* 9:88.

145. Shimbo K*, et al.* (2014) Exosome-formed synthetic microRNA-143 is transferred to osteosarcoma cells and inhibits their migration. *Biochem Biophys Res Commun* 445(2):381-387.

146. Kojima S*, et al.* (2014) The tumor-suppressive microRNA-143/145 cluster inhibits cell migration and invasion by targeting GOLM1 in prostate cancer. *J Hum Genet* 59(2):78-87.

147. Wu D*, et al.* (2013) MicroRNA-143 inhibits cell migration and invasion by targeting matrix metalloproteinase 13 in prostate cancer. *Molecular medicine reports* 8(2):626-630.

148. Zhong W*, et al.* (2013) [Expression of miR-143 in nasopharyngeal carcinoma cell lines and its effect on cell adhesion ability]. *Nan Fang Yi Ke Da Xue Xue Bao* 33(4):582-585.

149. Hu Y, Ou Y, Wu K, Chen Y, & Sun W (2012) miR-143 inhibits the metastasis of pancreatic cancer and an associated signaling pathway. *Tumour biology : the journal of the International Society for Oncodevelopmental Biology and Medicine* 33(6):1863-1870.

150. Zhang Y*, et al.* (2012) MicroRNA-143 targets MACC1 to inhibit cell invasion and migration in colorectal cancer. *Mol Cancer* 11:23.

151. Song T*, et al.* (2011) Expression of miR-143 reduces growth and migration of human bladder carcinoma cells by targeting cyclooxygenase-2. *Asian Pacific journal of cancer prevention : APJCP* 12(4):929-933.

152. Peng X*, et al.* (2011) Identification of miRs-143 and -145 that is associated with bone metastasis of prostate cancer and involved in the regulation of EMT. *PLoS One* 6(5):e20341.

153. Cimino D*, et al.* (2013) miR148b is a major coordinator of breast cancer progression in a relapse-associated microRNA signature by targeting ITGA5, ROCK1, PIK3CA, NRAS, and CSF1. *FASEB journal : official publication of the Federation of American Societies for Experimental Biology* 27(3):1223-1235.

154. Zhao G*, et al.* (2013) miR-148b functions as a tumor suppressor in pancreatic cancer by targeting AMPKalpha1. *Mol Cancer Ther* 12(1):83-93.

155. Fan Y*, et al.* (2014) miR-19b promotes tumor growth and metastasis via targeting TP53. *Rna* 20(6):765-772.

156. Wu Q*, et al.* (2014) MiR-19a/b modulate the metastasis of gastric cancer cells by targeting the tumour suppressor MXD1. *Cell death & disease* 5:e1144.

157. Zhang J*, et al.* (2012) miR-21, miR-17 and miR-19a induced by phosphatase of regenerating liver-3 promote the proliferation and metastasis of colon cancer. *Br J Cancer* 107(2):352-359.

158. O'Hara AJ*, et al.* (2009) Tumor suppressor microRNAs are underrepresented in primary effusion lymphoma and Kaposi sarcoma. *Blood* 113(23):5938-5941.

159. Shen S*, et al.* (2014) A prognostic model of triple-negative breast cancer based on miR-27b-3p and node status. *PLoS One* 9(6):e100664.

160. Ye J*, et al.* (2013) miRNA-27b targets vascular endothelial growth factor C to inhibit tumor progression and angiogenesis in colorectal cancer. *PLoS One* 8(4):e60687.

161. Ishteiwy RA, Ward TM, Dykxhoorn DM, & Burnstein KL (2012) The microRNA -23b/-27b cluster suppresses the metastatic phenotype of castration-resistant prostate cancer cells. *PLoS One* 7(12):e52106.

162. Syring I*, et al.* (2014) Circulating serum microRNA (miR-367-3p, miR-371a-3p, miR-372-3p, miR-373-3p) as biomarkers for patients with testicular germ cell cancers. *J Urol*.

163. Fang L*, et al.* (2013) Versican 3'-untranslated region (3'-UTR) functions as a ceRNA in inducing the development of hepatocellular carcinoma by regulating miRNA activity. *FASEB journal : official publication of the Federation of American Societies for Experimental Biology* 27(3):907-919.

164. Zhu X*, et al.* (2012) MTA1 gene silencing inhibits invasion and alters the microRNA expression profile of human lung cancer cells. *Oncol Rep* 28(1):218-224.

165. Yamamoto Y*, et al.* (2009) MicroRNA-500 as a potential diagnostic marker for hepatocellular carcinoma. *Biomarkers : biochemical indicators of exposure, response, and susceptibility to chemicals* 14(7):529-538.

166. Cha ST*, et al.* (2010) MicroRNA-519c suppresses hypoxia-inducible factor-1alpha expression and tumor angiogenesis. *Cancer Res* 70(7):2675-2685.

167. Katayama Y*, et al.* (2012) Identification of pathogenesis-related microRNAs in hepatocellular carcinoma by expression profiling. *Oncology letters* 4(4):817-823.

168. Li J*, et al.* (2010) An inhibitory effect of miR-22 on cell migration and invasion in ovarian cancer. *Gynecologic oncology* 119(3):543-548.

169. Lodewijk L*, et al.* (2012) The value of miRNA in diagnosing thyroid cancer: a systematic review. *Cancer biomarkers : section A of Disease markers* 11(6):229-238.

170. Guo M*, et al.* (2014) miR-656 inhibits glioma tumorigenesis through repression of BMPR1A. *Carcinogenesis* 35(8):1698-1706.

171. Rani S, Gately K, Crown J, O'Byrne K, & O'Driscoll L (2013) Global analysis of serum microRNAs as potential biomarkers for lung adenocarcinoma. *Cancer Biol Ther* 14(12):1104-1112.

172. Laddha SV*, et al.* (2013) Genome-wide analysis reveals downregulation of miR-379/miR-656 cluster in human cancers. *Biology direct* 8:10.

173. Dong Y*, et al.* (2011) MicroRNA dysregulation in colorectal cancer: a clinical perspective. *Br J Cancer* 104(6):893-898.

174. Wu WK*, et al.* (2011) MicroRNA in colorectal cancer: from benchtop to bedside. *Carcinogenesis* 32(3):247-253.

175. Lyu X*, et al.* (2014) TGFbetaR2 is a major target of miR-93 in nasopharyngeal carcinoma aggressiveness. *Mol Cancer* 13:51.

176. Wang L, Wang Q, Li HL, & Han LY (2013) Expression of MiR200a, miR93, metastasis-related gene RECK and MMP2/MMP9 in human cervical carcinoma--relationship with prognosis. *Asian Pacific journal of cancer prevention : APJCP* 14(3):2113-2118.

177. Fang L*, et al.* (2012) MiR-93 enhances angiogenesis and metastasis by targeting LATS2. *Cell Cycle* 11(23):4352-4365.
